# The Expanded Role of Chitosan in Localized Antimicrobial Therapy

**DOI:** 10.3390/md19120697

**Published:** 2021-12-08

**Authors:** Lisa Myrseth Hemmingsen, Nataša Škalko-Basnet, May Wenche Jøraholmen

**Affiliations:** Drug Transport and Delivery Research Group, Department of Pharmacy, UiT The Arctic University of Norway, Universitetsvegen 57, 9037 Tromsø, Norway; natasa.skalko-basnet@uit.no

**Keywords:** chitosan, antimicrobial activity, topical infections, antimicrobial resistance, drug delivery systems, scaffolds, skin infections, vaginal infections

## Abstract

Chitosan is one of the most studied natural origin polymers for biomedical applications. This review focuses on the potential of chitosan in localized antimicrobial therapy to address the challenges of current rising antimicrobial resistance. Due to its mucoadhesiveness, chitosan offers the opportunity to prolong the formulation residence time at mucosal sites; its wound healing properties open possibilities to utilize chitosan as wound dressings with multitargeted activities and more. We provide an unbiased overview of the state-of-the-art chitosan-based delivery systems categorized by the administration site, addressing the site-related challenges and evaluating the representative formulations. Specifically, we offer an in-depth analysis of the current challenges of the chitosan-based novel delivery systems for skin and vaginal infections, including its formulations optimizations and limitations. A brief overview of chitosan’s potential in treating ocular, buccal and dental, and nasal infections is included. We close the review with remarks on toxicity issues and remaining challenges and perspectives.

## 1. Introduction

The Organization for Economic Co-operation and Development has recently predicted that 2.4 million people in Europe, North America, and Australia will die from infections with resistant microorganisms in the next 30 years [1]. World Health Organization (WHO) considers increased antibiotic resistance the most significant challenge to human health [2]. In an era of increased incidence of resistant microorganisms (antimicrobial resistance, AMR) and alarming reports of confirmed resistance to most prescribed antibiotics [3], it is of tremendous importance to look at all available resources to combat this threat. Yet, surprisingly unsuccessful pipelines of antimicrobial candidates [4] indicate that our search might not be well-enough targeted to lead to fast and viable improvements.

Although a single approach cannot address such a complex and multi-causative threat, we propose that more attention be given to utilizing nature as a source of the safe and efficient antimicrobial arsenal. Despite the great potential of marine resources as biodegradable biomaterials and an increasing number of marine organism-based biomaterials reaching the market, the full potential of these resources is yet to be realized [5]. Among various natural antimicrobials, we aimed to highlight the pharmaceutical excipient that has not reached its full potential as antimicrobial, namely chitosan. 

Chitosan originates from chitin, the second most abundant polysaccharide extracted from the shells of crustaceans and cytoderm of fungi and green algae [6]. It has been proven to exhibit potent antimicrobial activities [7], and it can act in synergy to enhance the antimicrobial potential of other antimicrobials [8]. However, it has also been shown that the formulation features are critical for achieving the maximum effect of any antimicrobial [2] and thus need to be tailored when utilizing chitosan’s antimicrobial potential [8]. Moreover, its formulations in the nanosized range were reported to restrain the resistance development in bacteria [9]. 

### 1.1. Localized Therapy 

Localized drug therapy offers numerous advantages such as:

- Achieving a high drug concentration at the infection site,
- Limiting systemic drug exposure often responsible for faster development of AMR,
- Consequently, reducing the systemic side effects,
- Improving the safety drug profile in, for example, pregnant patients [10].

### 1.2. Structure of Review 

In the review, we offer a novel focus on the role of chitosan as an intrinsic antimicrobial. When facing the limited antimicrobial therapy options accompanied by a scarcity of novel antimicrobials, it is imperative to extend the arsenal of antimicrobial resources beyond current pipelines. Therefore, here we highlight the challenges of AMR and consider the advantages of efficient localized drug therapy. We focus on the extended role of chitosan as an excipient with intrinsic antimicrobial properties, which remain to be fully explored. We address the infection site’s challenges and the corresponding challenges the chitosan formulation should overcome to optimize the treatment. We also provide an extensive overview of representative literature, emphasizing the main antimicrobial findings and the delivery systems summary based on the type of targeted microorganisms. The in-depth review highlights the role of chitosan in skin and vaginal infections, with a brief overview of the role of chitosan in ocular, buccal and dental, and nasal sites. We finalize the review by addressing the toxicity, limitations, sustainability, and perspectives of chitosan’s antimicrobial use.

## 2. Polymers’ Role in Antimicrobial Therapy

### 2.1. Polymers Used in Localized Antimicrobial Therapy

As an alternative to traditional antibiotics, more emphasis could be placed on natural resources for drug delivery strategies [2]. Since around half of the earth’s biodiversity is found in the oceans, polymers from marine sources are particularly interesting in that respect [11,12].

Both natural and synthetic polymers are frequently used as excipients in biomedical applications intended for antimicrobial therapy. In recent years, the attraction towards natural polymers has grown due to the distinct properties and the abundance of these compounds in nature. Among the natural polymers with an inherent antimicrobial activity, chitosan appears as one of the most promising [13,14]. The synthetic polymers could also bear antimicrobial properties or could be modified to exhibit such properties. Antimicrobial polymers bearing quaternary ammonium or phosphonium as functional groups are the most widely studied among the polymers with antimicrobial properties [14,15]. The most frequently used synthetic polymers for localized therapy are poly(caprolactone) (PCL), poly(lactic acid) (PLA), poly(lactic-co-glycolic acid) (PLGA), and poly(vinyl alcohol) (PVA) [16]. Furthermore, these polymers are also used alone or in combination with other natural and synthetic polymers to improve the treatment outcomes or physical properties.

### 2.2. Chitosan’s Antimicrobial Properties and Its Role in Localized Antimicrobial Therapy

Chitosan is a natural polycationic linear polysaccharide composed of (1→4)-2-amino-2-deoxy-β-d-glucan and (1→4)-2-acetamido-2-deoxy-β-d-glucan obtained from deacetylated chitin [17,18,19]. Chitin is the second most abundant natural polysaccharide, whereas chitosan is the most investigated marine polysaccharide [11,17]. Chitin is not only commonly found in marine sources but also in fungi; however, its content there is lower than in crustaceans [20]. Nevertheless, with correct cultivation, fungi could be a realistic alternative source to the crustaceans for biomaterials and biomedical applications [21].

In addition to the intrinsic antimicrobial [22,23,24,25] and antifungal [26,27,28] activity of chitosan, this polymer exhibits anti-inflammatory activity [29,30,31], antioxidative effects [32,33], hemostatic activity [34,35], and healing properties [36,37,38]. Importantly, chitosan is both biocompatible [39,40] and biodegradable [41] and is approved by the U.S. Food and Drug Administration for certain applications [42]. These properties make chitosan an attractive building block in drug delivery systems and scaffolding. Furthermore, its muco and bioadhesive properties [43,44] increase its applicability in topical, local applications and formulations intended for skin and mucosal membranes. These properties are valuable attributes in materials intended to treat mucosa surfaces and infected, inflamed, and damaged skin. However, the biological activity of chitosan is dependent on the degree of deacetylation (DDA) and molecular weight (MW) [45]. Due to the deacetylation of the poorly soluble chitin, pH affects the activity and solubility of chitosan. The polycationic nature of chitosan is the basis for most of its biological activities [17]. In addition to effects of deacetylation, the chitosan’s functional groups, the amino- and hydroxyl- groups, are used as sites for polymer modifications to improve its physical and biological features [46].

The mechanisms behind the antimicrobial activity of chitosan are not clear; however, the four most prominent known mechanisms are: (i) electrostatic interactions with microbial membranes and disruption of the membrane, (ii) complexation with microbial DNA, (iii) accumulation and enveloping on the microbial membrane, and (iv) chelation of divalent cations [18,19].

Bacterial membranes are slightly negatively charged in Gram-negative bacteria mainly because of lipopolysaccharides in the outer membrane and in Gram-positive bacteria due to lipoteichoic acids [47]. Due to its polycationic nature, chitosan can interact with the bacterial membrane [19]. This interaction causes destabilization and changes the membrane permeability, leading to the lysis of the bacteria [48,49,50].

The second mechanism is a complexation between chitosan and DNA mainly linked to the chitosan of lower MW. Because of the complexation between chitosan and DNA, the bacterial protein synthesis is hampered [19], resulting in death in both bacteria and fungi [51,52].

Chitosan, especially of higher MW, can deposit on the bacterial membrane (due to the nature of the membrane) and create an envelope on the surface of the bacteria, disrupting the bacteria’s uptake of nutrients and waste removal, leading to bacterial death [18,53].

The fourth mechanism is based on chitosan’s ability to chelate divalent cations [54]. Bacteria utilize these divalent cations to stabilize the membrane; therefore, chitosan destabilises the bacterial membrane [18].

The antimicrobial mechanisms of chitosan against fungi and viruses are even less studied than those against bacteria. However, the antifungal activity is believed to originate from the interaction between chitosan and the negatively charged fungal membrane caused by the presence of negatively charged sialic acids [19,55]. The antiviral activity of chitosan might also originate in the electrostatic interactions with chitosan and subsequent direct eradication. It is also proposed that the activity might result from chitosan’s ability to block viruses from interacting with the cell surface or by boosting immune response [56].

## 3. Chitosan-Based Drug Delivery Systems for Localized Antimicrobial Therapy

### 3.1. Particles and Carriers

Chitosan-based microparticles (MPs) and nanoparticles (NPs), or carriers, have attracted attention as drug delivery systems in infection control and prevention due to their unique characteristics [57,58]. Generally, polymeric particles could protect the active compound from degradation, provide controlled release of the antimicrobial compound into the intended tissue, enhance penetration or permeation, increase the concentration at the intended site and intracellularly, and reduce adverse effects and systemic exposure [57,58,59,60]. Chitosan-based MPs and NPs are utilized in microbial eradication as carriers of antimicrobial compounds because of their inherent antimicrobial activity resulting from the high density of positively charged amino groups and the surface-to-volume ratio [60,61]. Furthermore, chitosan particles are considered safe to mammalian cells, able to modulate the immune system, and possess excellent permeability enhancing properties [62]. Chitosan particles alone [63], as well as several aspects of their antimicrobial activity, have been assessed, including acting as carriers of other antimicrobial compounds [64,65] and being embedded in a secondary system [66], all intended for localized, antimicrobial therapy.

### 3.2. Coating Material and Excipients

Chitosan offers versatile ways of application in pharmaceutical formulations. As coating material or excipient in drug delivery systems, chitosan can introduce beneficial qualities that tailor the system for topical application and optimize antimicrobial activity [67]. The addition of chitosan to a formulation intended for localized therapy can improve applicability by providing bioadhesiveness and sustained release to the system [68]. Moreover, the antimicrobial activity might be altered due to the intrinsic characteristics of chitosan or the synergy of polymer and active ingredients [69]. A variety of these strategies have been studied; chitosan-coating of MPs and NPs, chitosan-functionalized systems, encapsulated chitosan, hybrid systems including chitosan, and surface modification of drug delivery systems [8,22,70,71,72]. Moreover, the preparation method can tailor the properties of chitosan to improve the suitability of formulation for the specific routes of application [8,73].

### 3.3. Polymer-Based Gels

Chitosan-based hydrogels can be applied as therapeutic drugs vehicles or active ingredients, secondary vehicles for MPs and NPs containing drugs or active ingredients, and as intrinsic antimicrobials [67,74]. The use of chitosan-based hydrogels in pharmaceutical applications is widely studied, especially targeting improved wound healing and therapy of localized infections [75,76]. Numerous favourable properties of chitosan-based gels, such as biocompatibility, biodegradability, bioadhesiveness, antimicrobial activity, as well as the ability to disrupt biofilms, make them highly attractive for biomedical applications [74,77]. Moreover, chitosan-based hydrogels provide sustained and controlled drug delivery through various environmental stimuli, such as thermosensitive or pH-sensitive gels [67].

### 3.4. Scaffolds

There are several requirements involved in scaffolds intended for localized infections, and the specific requirements depend on the site of the infection and the condition of the infected area. Scaffolds must be biocompatible, biodegradable, maintain moisture balance, allow oxygen exchange, protect from the surrounding environment, and permit cell migration and tissue regeneration [78]. Scaffolds involving chitosan have shown the ability to maintain moisture balance [79], allow oxygen exchange [80], protect from the environment [81], allow cell migration [81], and promote factors important for tissue regeneration [82]. Among the scaffolds, sponges, films, fibres, and matrices are widely used [78].

**Figure 1:**
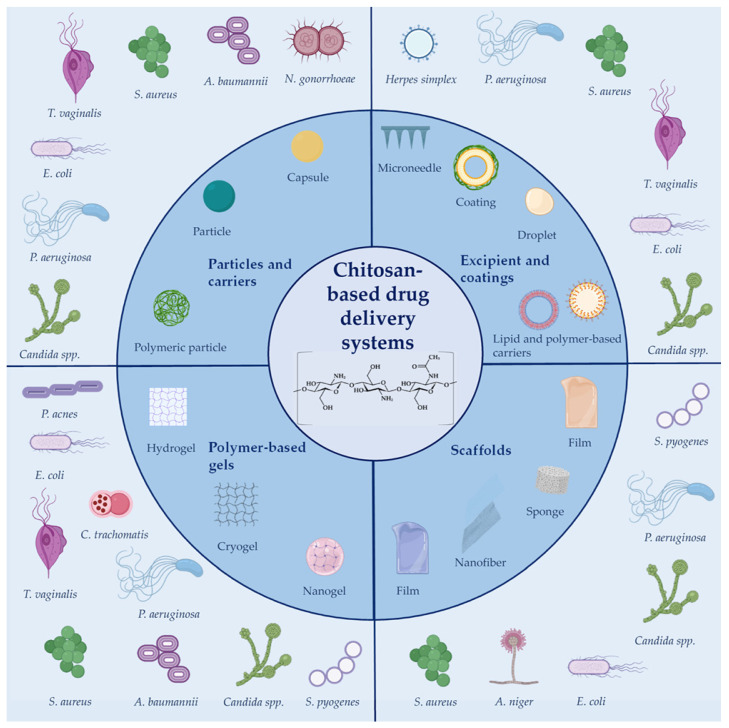
Summary of chitosan-based drug delivery systems and scaffolds with their respective targeted microorganisms. The illustration is created with BioRender.com.

Chitosan-based delivery systems for the localized therapy of infections are summarized in Figure 1.

## 4. Role of Chitosan in Localized Therapy of Skin Infections

### 4.1. Common Skin Infections and Microorganisms

The skin is the largest organ of the human body and is constantly in contact with its surroundings [83], serving as the body’s first defence line [84]. The skin is continuously challenged by essential, opportunistic, and pathogenic microorganisms that could cause infections if the skin barrier is breached or otherwise compromised [85,86]. Even with an effective protective barrier, infections are relatively frequent, becoming a growing concern [87]. The burden of skin infections covers a wide variety of conditions often classified as skin and soft tissue infections (SSTIs). Uncomplicated SSTIs are typically comprised of impetigo, ecthyma, erysipelas, and folliculitis, while complicated SSTIs include more severe, acute wound infections, chronic wound infections, cellulitis, and necrotizing skin infections [88]. U.S. Food and Drug Administration defines the latter as acute bacterial skin and skin structure infections [89]. Additionally, chronic wounds are considered a massive burden on the health care systems [90], with *Staphylococcus aureus*, *Pseudomonas aeruginosa*, and *Escherichia coli* among the bacteria most frequently found in these skin infections [91]. Wounds and other skin infections are also polymicrobial in nature, therefore, they are more complex and harder to treat [92].

Superficial fungal infections are an increasing problem affecting between 20–25% of the global population [93,94]. A fungus of importance is *Candida albicans*, an opportunistic fungus and a natural part of the human microbiota frequently found in microbial communities in chronic wounds [95,96]. Resistance among fungal species is growing, and relatively few treatment options are available [97].

Furthermore, there are many viral infections such as varicella-zoster, herpes zoster, molluscum contagiosum virus, and human herpesvirus 6. However, these infections are often self-limiting and do not require treatment [98]. Herpes simplex virus infections, such as herpes labialis, could be treated locally if the outbreak of the infection is localized in a limited area [99,100]. However, localized, antiviral skin therapy utilizing chitosan delivery systems is underrepresented compared to antibacterial and antifungal treatment.

The burden of infected nonhealing or hard-to-heal wounds, along with other skin diseases, is rapidly increasing, creating a strain on medical services [101]. The term chronic wounds covers a wide variety of conditions, with diabetic foot ulcers, venous leg ulcers, and pressure ulcers being the most relevant [102]. The majority of these wounds are halted in the inflammatory phase, which delays the proliferation and epithelization and decelerates the healing process [103]. Chronic wounds usually are polymicrobial in nature, with bacteria linked to the genetics of each patient [104]. However, *S. aureus* and *P. aeruginosa* are recognized as strong contributors to chronicity in nonhealing wounds [105]. Wolcott et al. analyzed bacteria found in chronic wounds of 2963 patients, and *S. aureus*, *Staphylococcus epidermidis, Finegoldia magna*, and *P. aeruginosa* were the most frequently found in these wounds [106]. Yet, the presence of certain microorganisms in the wound bed does not directly indicate an infection [107]. *S. aureus* and *P. aeruginosa* are the most common bacteria to produce biofilm networks in the wound bed [103,108], and these biofilms are communities of bacteria living together in extracellular polymeric substances which provide protection and facilitate adhesion to the affected tissue [109]. In addition to the increased protection of microorganisms in biofilms, the matrix allows improved communication between bacteria and increases resistance by allowing bacteria to remain in a dormant state for prolonged periods [103,107]. These biofilm matrices are found in approximately 60–80% of all human infections [110], and bacteria in biofilms are often 1000-fold more resistant than planktonic bacteria, further increasing the challenge in eradicating these pathogens [111]. Fungi are also often a part of the polymicrobial community in chronic wounds, and *Candida* spp. are regularly reported as the most recurrent fungi [95]. Wounds or other skin breaches could potentially cause other forms of SSTIs if left untreated [112]. Utilizing topical antibiotics in the treatment of chronic wounds is not uncommon; however, the evidence behind their use is somewhat limited [113]. The selection of the type of antibiotic is dependent on the microbial picture [105,110].

### 4.2. Challenges of Antimicrobial Treatment and Delivery to the Skin

The skin represents an attractive route in the therapy of localized microbial infections, mainly due to the potential of higher local drug concentrations [114]. Additionally, the demand for therapeutic options for skin infection is increasing with growing numbers of SSTIs globally [115]. However, several limitations are linked to the skin as a target site for delivering antimicrobial compounds.

The skin structure comprises three main layers; epidermis, dermis, and hypodermis [83]. The epidermis mainly consists of keratinocytes and is where the binding of pharmaceutical compounds, metabolism, and active transport occurs [116]. Below the epidermis is a layer of connective tissue composed of fibroblasts, namely the dermis, responsible for the structure and elasticity of the skin [117]. The hypodermis, or the subcutaneous tissue, is the innermost layer of the skin and consists of connective tissue, however, looser than the connective tissue in the dermis. The main functions of the hypodermis are protection from physical impact, temperature regulation, and energy storage [117]. The uppermost and primary protective layer, the *stratum corneum* (SC), plays a key role in dermal delivery [118]. The SC limits the penetration of active compounds due to the lipophilic nature of the barrier, which prevents absorption of molecules with an MW of more than 500 Da [118,119,120]. The SC is structured in a distinct way, often referred to as the brick-and-mortar model, where the corneocytes represent the bricks and the lipid matrix represents the mortar [83]. In the localized treatment of dermal infections, the aim is to assure penetration into the infection site while avoiding the potential absorption. The penetration of the active compound might be slow, creating challenges in reaching the desired concentration [121]. Determining the concentration of the compound within the skin layers, as well as determining the compound clearance and degradation from these layers, is challenging as well as [122].

In localized skin infections, the heterogeneity between patients might pose another challenge. The skin condition might be altered if the patient suffers from SSTIs and skin impairments in general. In the case of wounds or impaired skin, the skin’s barrier function might be lost, and penetration or permeation through the skin might increase [83]. This might lead to lowered concentrations of the antimicrobial compounds in the intended site and could increase the potential of systemic side effects. Furthermore, the skin or wound environment might be altered, and the skin barrier weakened because of, and depending on, the given condition, e.g., infection, wounds, lesions, or inflammation, or thickened, due to ichthyosis or cancer. In these cases, the absorption into or through the skin is different from normal or healthy skin [123]. Additionally, impairment of the skin, especially if the damage reaches into the deeper skin layers or is bleeding, could cause alteration to the pH on the surface from the naturally more acidic milieu on the SC. This more neutral environment is beneficial for the growth of many pathogenic microorganisms, further increasing the challenge in treating the SSTIs [124]. Impaired skin, especially wounds and lesions, could contain high volumes of exudate, essential for maintaining a proper healing cascade and maintaining a moist wound bed. However, extensive volumes of exudate could lead to further reduced healing [125,126] and introduce challenges in maintaining contact between therapeutic formulation and the infection site, leading to lower retention or residence time of the therapeutic in the affected area [107]. These and other factors, such as low permeation or degradation, could result in the need for frequent drug administration onto a damaged and painful area [127] and further impaired healing [127]. In addition, the potential of local side effects or skin irritation needs to be accounted for [114]. Allergic contact dermatitis is not uncommon [118]. Occlusion effects could also potentially increase pH and temperature at the treatment site and cause skin irritation; however, these effects are often easy to avoid [118].

The most significant challenges in antimicrobial skin therapy are the numerous variations in conditions and the microbial diversity between patients and within the same SSTI [83,113]. The microbial picture depends on the patients’ genetics and the environment and varies between different sites on the human body [104,128]. Furthermore, biofilms can be found in up to 80% of all infections [110]. The AMR results from different mechanisms, such as restricted penetration of antimicrobial compounds into the biofilm matrix, altered metabolic activity in the microorganisms, altered gene expression, and microorganisms that remain in a dormant state. Topical therapy might increase the local concentration of antimicrobial compounds, such as mupirocin, metronidazole, and silver sulfadiazine [129]. Moreover, the biofilms are often firmly adhering to skin structures and might stretch further into the deeper skin layers, rendering removal challenging [107,125].

### 4.3. Tackling the Challenges of Infected Skin—The Delivery Strategies, Systems, and Scaffolds

In SSTIs and wound healing, chitosan is utilized to produce various drug delivery systems and scaffolds for wound healing and tissue regeneration. The range of these systems and scaffolds varies between polymer-based gels, nanofibrous scaffolds, and particles for regeneration, antimicrobial activity, and carrying the antimicrobial compounds. However, most systems and scaffolds are intended to heal wounds [78].

The scaffold should protect the wound from external contamination, maintain moisture, allow gas exchange, prevent bacterial growth, or eradicate bacteria in the wound bed, facilitate all stages of the wound healing cascade, and it should be biodegradable and biocompatible [62,78,130]. Chitosan possesses many of these necessary properties [16,19]. Additionally, it can assist in a sustained and controlled release of pharmaceutical compounds, such as antimicrobials, both in micro and nanotechnology [130,131,132]. Its retention in the skin could be improved while maintaining the balance between retention and penetration to ensure sufficient concentrations of the pharmaceutical compound [133]. Furthermore, advanced scaffolds could mimic the extracellular matrix to further promote skin healing [130].

**Figure 2:**
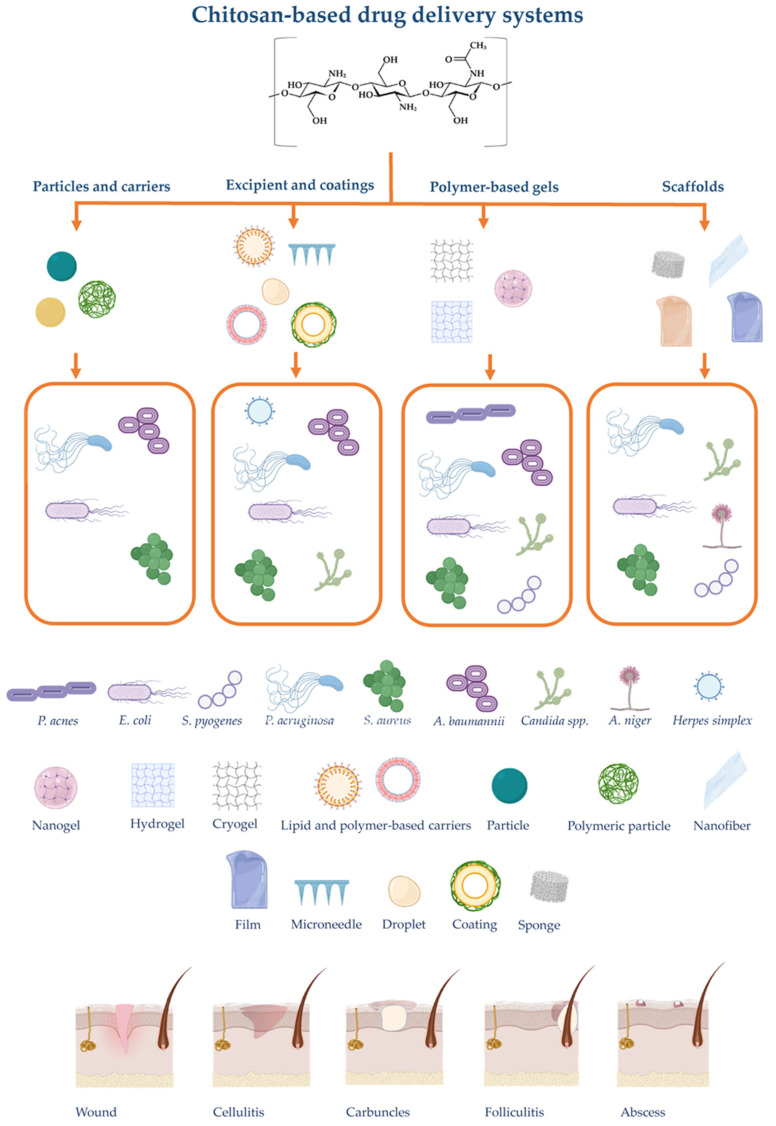
Summary of chitosan-based drug delivery systems and scaffolds intended for skin administration with their respective targeted microorganisms and examples of skin infections. The illustration is created with BioRender.com.

These delivery systems could be classified according to composition and size range. In skin repair and wound healing, these could be classified as NPs, nanocomposites, coatings, and scaffolds, depending on their intended use and attribution in the therapy [62]. As a bioactive polymer, chitosan is easily developed for gels, membranes, nanofibers, MPs, NPs, sponges, and scaffolds [134]. Chitosan-based delivery systems targeting skin infections are summarized in Figure 2.

#### 4.3.1. Particles and Carriers

Chitosan microparticles (CMPs) and nanoparticles (CNPs) are often used as carriers in skin therapy and especially in wound healing [135]. These drug delivery systems could provide prolonged and controlled release, protect the drug from degradation, reduce the administration frequency, and solubilize the antimicrobial compounds [135]. These particles and carriers could also display antimicrobial and anti-inflammatory properties [135]. Costa et al. prepared CNPs through ionic gelation with tripolyphosphate and challenged their in vitro activity against biofilm formation in vancomycin-resistant *S. aureus* (VRSA), vancomycin-resistant *Enterococcus faecalis*, and *P. aeruginosa* and *Acinetobacter baumannii*, in addition to the clinical strains of multiresistant *A. baumannii* and resistant *P. aeruginosa*. The CNPs inhibited biofilm formation in all strains; however, the effect was reduced in VRSA compared to the other strains. Resistant strains of *A. baumannii* and *P. aeruginosa* were more sensitive to the CNPs than the nonresistant strains [136]. In another study, Hajji et al. evaluated the antimicrobial activity of CNPs loaded in gelatin/chitosan films against *Bacillus cereus*, *S. aureus*, *Micrococcus luteus*, *E. coli*, *Klebsiella pneumoniae*, *Salmonella enterica*, *Salmonella typhimurium*, and *Enterobacter* spp. bacterial strains. The films loaded with CNPs exhibited higher antimicrobial activity in a concentration-dependent manner against most strains, especially Gram-positive strains, compared with the unloaded films [137]. Furthermore, Vila-Sanjurjo et al. tailored genipin and tripolyphosphate dual crosslinked CNPs and demonstrated quorum quenching activity in *E. coli*. Blocking the quorum sensing activity could potentially improve the antibiofilm effect of this type of drug delivery system [138].

In addition to being utilized for their intrinsic antimicrobial activity, CNPs are used as carriers for both conventional and traditional antimicrobial compounds from natural sources such as plants. Curcumin is a polyphenol utilized in wounds because of its antibacterial, anti-inflammatory, and antioxidative effects [139]. Due to the limited solubility and bioavailability of curcumin, CNPs have been utilized to improve curcumin’s antimicrobial and wound healing properties [65,140]. Basit et al. proposed curcumin-CNPs against two common skin pathogens, namely *S. aureus* and *P. aeruginosa*. The authors demonstrated concentration-dependent antibacterial activity from both curcumin-containing and curcumin-free CNPs in both strains; however, curcumin-containing CNPs displayed superior activity [140]. Similarly, Saranya et al. utilized CMPs conjugated with curcumin against *S. aureus* and *E. coli* to prevent skin infections. The CMPs with curcumin had an increased inhibition zone and improved inhibition in serial dilution than curcumin alone in both strains [65].

Along with curcumin, other polyphenols have been utilized to treat skin infections. Fras Zemljič et al. prepared CNPs and CMPs with catechin and resveratrol to enhance microbial eradication and improve wound healing. These particles were embedded in poly(ethylene oxide) (PEO) nanofibers and challenged against *S. aureus* and *E. coli*. The reported reduction of bacteria was over 83 and 99% for *S. aureus* and *E. coli*, respectively [141].

Other compounds from natural sources were also used in CMPs and CNPs. Propolis-CMPs inhibited biofilm formation and improved eradication of preformed biofilm in *E. faecalis* [142], melatonin loaded in chitosan/poloxamer NPs demonstrated antibiofilm activity against *S. aureus.* The methicillin-resistant *S. aureus* (MRSA) [143], essential oil of *Homalomena pineodora* in CNPs, generated greater inhibition zones in a range of Gram-positive and Gram-negative bacteria as well as yeasts [144], and CNPs with *Henna* extract improved antimicrobial activity against *S. aureus* and *C. albicans* [145].

The proteins, peptides, and compounds generating reactive oxygen species were also used as antimicrobial compounds in topical skin infections. Aramwit et al. compared the antimicrobial activity of sericin-loaded CNPs and a commercially available silver wound dressing in several Gram-positive and Gram-negative bacteria. In the Gram-positive strains, the sericin-loaded CNPs exhibited superior antibacterial activity [146]. Antimicrobial peptides are considered promising compounds as a solution to AMR challenges. Peptides from both cathelicidin and temporin families have been incorporated into CNPs [64,147,148]. OH-CATH30 loaded in carboxymethyl CNPs displayed strong antibacterial activity against *E. coli* [148], and LL-37 loaded in CNPs demonstrated complete bacterial eradication after seven days in a MRSA-infected wound model in mice [64]. Similarly, temporin B loaded in CNPs exhibited superior antibacterial activity against both a reference strain and clinical strains of *E. epidermidis* [147].

The compounds that generate reactive oxygen species, such as photodynamic agents, are considered promising in AMR [149]. Utilizing photodynamic therapy, indocyanine green was entrapped in CNPs and challenged against *A. baumannii* in a biofilm model. The treatment was superior to all other therapies against the biofilm [150]. Biofilms create substantial challenges in treating infected skin and especially wounds; thus, several studies on the antibiofilm effects of chitosan-based delivery systems and scaffolds are summarized in Table 1.

Chitosan-based MPs and NPs have also been utilized to improve the activity of conventional antimicrobial compounds. Silver sulfadiazine, commonly used in localized skin infections, has been incorporated in both MPs and NPs [167,168,169]. Silver sulfadiazine loaded in CNPs used as a coating on cotton gauzes displayed antimicrobial activity against *Bacillus subtilis*, *S. aureus*, *E. coli*, *P. aeruginosa,* and *C. albicans* [167], and silver sulfadiazine loaded in CMPs embedded in a PEGylated fibrin gel demonstrated antibacterial activity *S. aureus* and *P. aeruginosa* under in vitro conditions [169] as well as a superior antibacterial activity after 11 days in a *P. aeruginosa*-infected porcine model [168].

Cerchiara et al. reported the superior antibacterial effect of vancomycin loaded in CNPs embedded in Spanish Broom fibres compared with vancomycin alone against *S. aureus* [170]. Erythromycin loaded in CNPs embedded in cellulose acetate nanofibers similarly demonstrated a more significant inhibition zone in *S. aureus*, *E. coli*, and *P. aeruginosa* compared with the erythromycin in cellulose acetate nanofibers [171]. Niamlang et al. showed inhibition of *S. aureus*, *E. coli*, and *E. faecium* within 24 h upon embedding tetracycline-loaded CNPs in PVA films [66].

Various chitosan-based particles are used to incorporate conventional antifungal compounds, whereas the use of these particles to include antiviral compounds is scarce. Recently, Khalid et al. utilized NPs comprising chitosan and chondroitin sulfate as a delivery system for fluconazole aimed for dermal application against *C. albicans*. With these fluconazole-loaded CNPs, the burden of *C. albicans* was significantly reduced compared with the fluconazole alone, with approximately a 100-fold reduction of the yeast count [172]. Donalisio et al. tailored nanospheres with chitosan, mineral oil, dodecanol, and surfactants loaded with acyclovir against the *Herpes simplex* virus. Compared with acyclovir alone, the loaded nanospheres had higher antiviral activity against both strains [99].

#### 4.3.2. Coating Material and Excipients

Chitosan is often used to improve the biological or physical properties of the formulation. A broad range of research includes chitosan as an excipient or as a coating material for formulations intended for the treatment of skin infections. For example, chitosan was exploited for its bioadhesive properties as a lipid–polymer hybrid carrier of tea tree oil to improve wound healing. The antimicrobial activity of this formulation was further tested against *P. aeruginosa*, *S. aureus*, MRSA, and *C. albicans*. The hybrid carriers did not impede the activity of tea tree oil, and neither was it improved by the inclusion of the polymer [173].

The chitosan-shelled/decafluoropentane-cored oxygen-loaded nanodroplets were tailored to improve microbial eradication in chronic wounds. Both oxygen-loaded and unloaded nanodroplets significantly inhibited *C. albicans*; however, inhibition of MRSA was only observed during the first 4 h [174].

Rosmarinic acid loaded in chitosan encapsulated graphene NPs were challenged against *S. aureus*. The minimum inhibitory concentration (MIC) of the loaded NPs was found to be 0.00681 mg/mL, while the MIC of rosmarinic acid and chitosan was 0.8 and 0.08 mg/mL, respectively. The zone of inhibition was also improved in the NP formulation (13.3 mm) compared to the rosmarinic acid (12.4 mm) and chitosan (8.7 mm) after 48 h [175].

In another study, the beads comprising PVA and chitosan were utilized to incorporate zinc oxide NPs. Alongside improved healing rates in a murine model, the in vitro antimicrobial activity of the zinc oxide-loaded beads was superior to unloaded beads and chitosan [176].

Zan et al. utilized chitosan/poly(ethylenimine) microneedles loaded with amphotericin B against fungal skin infection. The microneedles were challenged in *C. albicans*-infection murine model and reduced fungal burden [177]. In microneedle systems, chitosan could be utilized in the internal segment of the delivery system, as exemplified by Permana et al. In this microneedle system, chitosan was used as NPs or coating on PCL, MPs, PCL, or PLGA with NPs further embedded in PVA/poly(vinyl pyrrolidone) (PVP) microneedles. The MPs were loaded with silver NPs [178], while the NPs were loaded with doxycycline as antimicrobial compounds [153]. For the microneedle system loaded with silver NPs, the antibiofilm activity was evaluated in an ex vivo biofilm model with *S. aureus* or *P. aeruginosa*. Here, the silver NPs loaded in MPs embedded in microneedles eradicated 100% of the biofilm for both strains and showed superiority to all tested formulations [178].

Several studies utilized chitosan as a coating, especially for NPs. Azzazy et al. utilized chitosan-coated PLGA NPs loaded with *Peganum harmala* alkaloids for antibacterial properties and wound healing. In this study, the chitosan-coated NPs were challenged against *S. aureus* and *E. coli* using in vitro broth dilution method. The loaded chitosan-coated NPs improved the antibacterial potential against *E. coli* and *S. aureus* [179]. In another research effort, chitosan-coated NPs comprising PLGA and PVA loaded with benzalkonium bromide were evaluated in an in vitro biofilm assay and an in vivo murine MRSA-infected wound model. The coated NPs displayed a significantly improved inhibition of biofilm formation compared with free benzalkonium bromide, as well as a reduced bacterial burden in the in vivo wound model [180].

Metal-based NPs are also frequently seen in delivery systems intended to eradicate microorganisms in skin and wounds. Daghian et al. designed a hybrid drug delivery system comprising silver and talc capped with chitosan to improve the healing of infected wounds. The authors tested the MIC and minimum bactericidal concentration (MBC) against *S. aureus*, *P. aeruginosa*, *Streptococcus pyogenes,* and *E. coli*. Additionally, the hybrid system was challenged against a murine *P. aeruginosa* and *S. aureus*-infected wound model and compared to mupirocin ointment. In the in vivo wound challenge, the bacterial burden in the mice treated with either mupirocin or the chitosan-capped hybrid system was lowered compared with the control noncapped hybrid system and talc. After 14 days, all other groups, except the control, had an extensive reduction of the bacterial burden [181]. The in vivo studies are important as a translational step to more marketed products and the use of chitosan in the fight against AMR. Therefore, studies with in vivo data on chitosan-based delivery systems and scaffolds are summarized in Table 2. In another research effort by Verma et al., tailored sericin and chitosan-capped silver NPs also improved wound healing and antimicrobial activity. Additionally, the capped silver NPs embedded in hydrogel demonstrated improved wound closure compared to a marketed povidone–iodine ointment and no dermal irritation [182]. The chitosan-capped copper oxide and copper NPs were prepared as composite films and challenged against *E. coli* and *Bacillus*. All formulations exhibited antimicrobial activity against both strains [183].

In addition to these inorganic materials, chitosan could be utilized as a coating for lipid-based systems, such as liposomes. This approach was used by Alshaman et al., where dicloxacillin was incorporated in chitosan-coated liposomes to improve the eradication of MRSA. The drug-loaded chitosan-coated vesicles were able to reduce the burden, whereas drug-free chitosan-coated and noncoated liposomes did exert some antimicrobial activity [203].

In addition, other oil-based formulations could also be used. Kumari and Kesavan evaluated clotrimazole-loaded microemulsions comprising clove oil, Tween 80, and propylene glycol coated with chitosan to improve the therapy of superficial fungal infections. The microemulsion system was challenged against *C. albicans.* Both the coated and noncoated microemulsions displayed antimicrobial properties. Furthermore, the authors evaluated skin retention of the coated and noncoated microemulsion, and these results revealed that almost 70% clotrimazole from the coated microemulsion remained in the skin after eight hours, while less than 40% remained after administering the noncoated emulsion. Retention in the skin is essential for topical therapeutical delivery to the skin [204].

Sandri et al. and Sun et al. combined chitosan with montmorillonite to increase antimicrobial eradication in the skin and wound infections [205,206]. The MBC of silver sulfadiazine loaded into the chitosan-containing composite against Gram-negative *E. coli* and *P. aeruginosa* was lower compared with the composite without chitosan [205]. The bacterial burden was lowered in an in vivo murine *S. aureus*-infected wound model upon treatment with 5-fluorocytosine loaded in chitosan-containing composite [206]. Furthermore, as an excipient, chitosan has been combined with nanofibers [207,208,209], gauzes [36,189], graphene quantum dots [186], and as a powder [210] or matrix for immobilization of enzymes [165,211] to improve antimicrobial properties and wound healing.

#### 4.3.3. Polymer-Based Gels

The gels and hydrogels are frequently used both as antimicrobial vehicles and drug delivery systems in localized drug therapy. In addition to serving as a drug carrier with prolonged or controlled release, gels maintain the moisture balance in skin lesions and wounds and provide bioadhesive properties to ensure retention at the intended site [212]. The cryogels are frequently developed as drug delivery systems for microbial eradication and treatment of skin infections. Due to the macroporous structure, they are both flexible and elastic and could improve the wound healing process [213]. Bölgen et al. applied this strategy to enhance the eradication of a broad range of microorganisms commonly found on the skin. The authors prepared *Hypericum perforatum* oil-loaded chitosan cryogels crosslinked with glutaraldehyde and further evaluated the antimicrobial potential of the delivery system using the disc diffusion method. The results revealed antimicrobial activity against all strains, namely *Enterococcus hirae*, *B. cereus*, *S. aureus*, *E. coli*, *Legionella pneumophila* subsp. *Pneumophila*, *P. aeruginosa*, and *C. albicans*, in a concentration-dependent manner. Additionally, the oil-loaded cryogel displayed an antioxidative effect and could potentially serve as a free radical scavenger [213].

In another research effort, Hou et al. tailored cryogels comprising glycol chitosan methacrylate and ε-poly-lysine acrylamide to improve eradication of resistant *S. aureus* in the wound bed. In the in vitro studies, the cryogel eradicated >99% of MRSA and *E. coli*, while in the in vivo MRSA-infected wound model, the mice treated with cryogel showed no signs of infection and improved wound closure [214]. Han and colleagues prepared cryogels combining chitosan and silk fibroin for improved mechanical strength. They used polydopamine NPs as near-infrared absorbing agents to improve antimicrobial properties and modulate the wound healing process. The cryogel with NPs, along with near-infrared irradiation, exhibited superior antibacterial properties, especially in the elevated NP concentrations. Furthermore, the cryogel with NPs also displayed superior anti-oxidative properties [215].

Aerogels are not as commonly used in antimicrobial eradication in skin infections and wound healing. Nevertheless, López-Iglesias et al. developed a novel chitosan aerogel loaded with vancomycin using a jet cutting technique. The antibacterial activity of the aerogel was evaluated using *S. aureus.* The aerogel demonstrated antibacterial activity and facilitated complete eradication in six hours, with the effect lasting for two days. Additionally, the aerogel could absorb nine times of its weight moisture in the wound bed [216].

Among the polymer-based gels, hydrogels are most frequently used in topical, antimicrobial skin therapy. Hydrogels provide good water balance in the wound bed, control the release of antimicrobial compounds, and allow oxygen exchange [19,217]. Ouyang et al. incorporated a marine peptide from seawater cultured tilapia in chitosan hydrogels. In the in vitro antimicrobial evaluations, the peptide-loaded hydrogel inhibited both *S. aureus* and *E. coli* [218].

More conventional antimicrobial compounds have also been loaded into chitosan hydrogels. Gentamicin-conjugated chitosan hydrogel was tailored as a scald dressing. In an in vitro disc diffusion assay, the gentamicin-grafted chitosan hydrogel displayed significant inhibitory activity against both strains compared to chitosan alone. In *S. aureus*, the inhibitory activity was superior to gentamicin alone [219].

El-Kased et al. proposed comparing polyacrylic acid and chitosan hydrogels as delivery systems for honey. Honey was loaded into the hydrogels, and antibacterial properties were assessed against *P. aeruginosa*, *S. aureus*, *S. pyogenes*, and *K.* *pneumonia*. In vitro disc diffusion tests proved antimicrobial activity from both chitosan and polyacrylic acid; however, the antimicrobial activity from hydrogels with chitosan was superior, and the activity increased with increasing honey concentrations. Only moderate activity was observed from the honey alone. In the in vivo murine burn wound model, no bacterial growth occurred in the noninfected wounds [220].

Along with studies on the antibacterial activity of chitosan hydrogel, studies on the antifungal activity have been performed. Özcan et al. incorporated terbinafine in chitosan hydrogels containing chitosan of different MW. All chitosan hydrogels loaded with terbinafine displayed antimicrobial activity against a wide range of filamentous fungi and *Candida* spp. Chitosan hydrochloride hydrogel exhibited the strongest inhibitory effect along with the fastest terbinafine release. The antimicrobial activity was superior to the action of a marketed product and similar to free terbinafine. In general, lower MW corresponded with higher antifungal effects [221].

To improve the responsiveness of the hydrogels, researchers have proposed using crosslinkers to tailor pH or thermal responsiveness in the hydrogel network [162,222,223]. To prepare pH-responsive hydrogel, designed for the altered pH in the wound bed, Heimbuck et al. utilized a combination of chitosan and genipin. Two chitosans with different MW were used in the evaluation, namely 15 and 50−190 kDa. In the antimicrobial evaluation of the hydrogels, the activities of two chitosan hydrogels were compared to chitosan films in *E. coli*. The films reduced the bacterial load by approximately 90%, while the chitosan-genipin hydrogels reduced growth by approximately 70%; no differences were observed between chitosan with lower and higher MW [222].

Introducing thermal responsiveness into the hydrogel network, researchers have utilized the crosslinker β-glycerolphosphate, as exemplified by Pati et al. [162] and Rezaei et al. [223]. Pati and colleagues proposed thermal responsive hydrogel loaded with 0.5 or 1% (*w/v*) ε-poly-L-lysine in the eradication of monomicrobial and polymicrobial biofilms in wounds produced by clinical isolates. In the in vitro monomicrobial biofilms, ε-poly-l-lysine loaded hydrogels eradicated >99% of *P. aeruginosa* and >70% of MRSA, while no antimicrobial activity was observed against *C. albicans*. In the ex vivo polymicrobial biofilm model, the loaded hydrogels significantly reduced the thickness of the biofilm and reduced the bacterial load of *P. aeruginosa* compared with the untreated biofilm; however, no reduction was observed for MRSA or *C. albicans*. The authors postulated that some bacteria were observed on the side of the biopsy and were therefore not in contact with the hydrogel [162]. Rezaei et al. prepared thermal responsive hydrogel to load the antimicrobial peptide Piscidin-1 to improve the eradication of clinical isolates of resistant *A. baumannii*. The standard strain of *A. baumannii* was inhibited by hydrogels loaded with 4 µg/mL of the peptide, while the clinical isolate required loading of 16 µg/mL before an inhibition zone was observed [223].

Chitosan hydrogels are often criticized for their mechanical properties. Introducing additives or combining chitosan with other polymers might improve those mechanical properties [20]. Masood et al. combined chitosan with polyethene glycol (PEG) in hydrogels loaded with silver NPs. Here, PEG served as a stabilizing agent in the hydrogel to increase swelling abilities and structure. The investigations of the antimicrobial activity against *E. coli*, *P. aeruginosa*, *B. subtilis,* and *S. aureus* showed that the silver NP-loaded hydrogels exhibited improved activity compared with silver NPs alone and chitosan hydrogel. The silver NP-loaded hydrogel also exhibited improved in vivo wound healing in a rabbit wound model with 48% wound closure after only four days [224].

**Figure 3:**
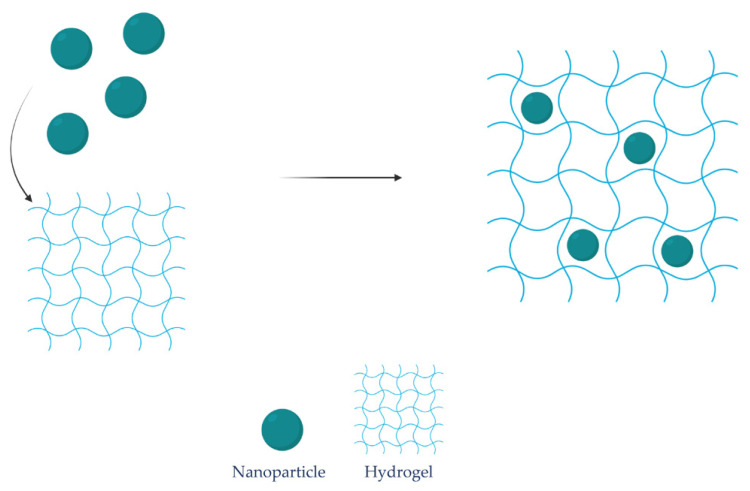
Concept of the primary and secondary vehicles to avoid some limitations of hydrogels represented as nanoparticles (primary) in hydrogel (secondary). The illustration is created with BioRender.com.

Another potential drawback with hydrogels is burst release from the hydrogel network. This drawback could be contacted by using a primary vehicle in the hydrogel, such as lipid-based vesicles, NPs, or other carriers (Figure 3). Additionally, these vesicles could improve the release profile of the delivery system [225]. Utilizing soy lecithin, Hemmingsen et al. prepared chlorhexidine-loaded liposomes embedded in chitosan hydrogel intended to treat chronic wounds. The antibiofilm activity of the loaded liposomes in the hydrogel was evaluated against *S. aureus, P. aeruginosa,* and a clinical isolate of *S. aureus*, where both the inhibition of biofilm formation and eradication of preformed biofilm were tested. The dual drug delivery system almost completely inhibited the formation of biofilm in both *S. aureus* and *P. aeruginosa*. Furthermore, the dual delivery system displayed strong in vitro anti-inflammatory properties [226].

Similarly, Sohrabi et al. prepared moxifloxacin-loaded niosomes embedded in LMW or MMW chitosan hydrogels. The antimicrobial activity of the dual delivery system comprising the medium MW chitosan was evaluated against *S. aureus* and *P. aeruginosa*. The niosomes appeared to improve the antimicrobial activity of moxifloxacin against *P. aeruginosa*; however, the smallest inhibition zones in both strains were observed for the dual system. On the other hand, the MIC values for the dual system and the moxifloxacin-loaded hydrogel were lowered in *S. aureus* compared to the different formulations and free drugs [227]. The strategy of utilizing vesicles embedded in hydrogels has also been tested against fungi, as in the work of AbdelSamie and colleagues [187].

#### 4.3.4. Scaffolds

Among the scaffolds, films and membranes are among the most common in the therapy of skin infections and infected wounds. These films have some beneficial properties, making them suitable for use in skin lesions and wounds. They allow for oxygen exchange and moisture evaporation simultaneously as they protect the area from contamination of bacteria from the external environment. Additionally, they are very flexible, allowing for easy application onto the skin and adaptation to the specific area of the body. Furthermore, they protect the incorporated compound until it is released into the intended area [228]. Pereira dos Santos and colleagues incorporated clove or melaleuca essential oils into chitosan films aimed at wound healing applications and eradicating bacteria and yeast. The authors evaluated the in vitro antimicrobial activity of the emulsion of the film composition against *S. aureus*, *E. coli* and *C. albicans*. The inhibition was lowered upon treatment with film emulsion compared to the essential oils alone. However, chitosan alone displayed antimicrobial activity against all strains [229]. In another study by Altiok et al., using thyme oil in chitosan films, the inhibition zone obtained in *S. aureus*, *P. aeruginosa*, *K. pneumoniae*, and *E. coli* were more significant. The chitosan films without oil did not have any antimicrobial effect [230]. Altiok and colleagues used higher chitosan concentrations and LMW chitosan, while Pereira dos Santos and colleagues used MMW chitosan. The DDA was approximately the same in these studies.

Other antimicrobial compounds from natural sources are also utilized in chitosan films for skin infections, such as capsaicin and curcumin. Akyuz and colleagues incorporated capsaicin into chitosan films with glycerol as a plasticizer and assessed the antimicrobial of three different concentrations in three Gram-positive and six Gram-negative strains. The loaded film showed antibacterial activity against all strains but seemingly higher activity against Gram-negative bacteria. Additionally, the loaded film displayed antiquorum sensing activity in *Chromobacterium violaceum* [231]. Upon loading with curcumin, Muthulakshmi and Rajarajeswari combined chitosan and pectin in the films. These films were assessed against *S. aureus* and *E. coli* and showed antibacterial activity against both strains. However, no activity was observed for curcumin-free films [232].

Chitosan films were also combined with more conventional antimicrobial compounds to treat skin and wound infections. Kausar and colleagues combined chitosan films with vancomycin to eradicate MRSA in burn wounds. The chitosan film with the highest vancomycin concentration (20%) showed the strongest antibacterial effect in a disc diffusion assessment compared with blank chitosan film, films loaded with lower concentrations of vancomycin (10%), and free drugs. Similar results were obtained in microdilution tests. In an in vivo rat wound model, the group treated with vancomycin-loaded films displayed the fastest wound healing with no detected bacterial growth [233].

Bavarsad et al. utilized griseofulvin to treat superficial fungal skin infections. Griseofulvin was loaded in liposomes embedded in chitosan films. These films were evaluated against *Microsporum gypseum* and *Epidermophyton floccosum* and showed antimicrobial activity in both strains, exhibiting the highest permeation across mice skin [234].

Ambrogi and colleagues combined montmorillonite with chitosan in composite films and utilized chlorhexidine diacetate as an antimicrobial compound. The film was challenged against *S. aureus*, *S. epidermidis*, *P. aeruginosa*, and *C. albicans*. All chlorhexidine films displayed antimicrobial activity in all strains; however, the activity was stronger in Gram-positive bacteria. Plain chitosan films had a moderate effect on the Gram-positive bacteria and *C. albicans*. The composite film without chlorhexidine had no activity in any of the strains. Almost all composite films loaded with chlorhexidine had a superior antibiofilm activity [235].

Silver sulfadiazine is, as mentioned, a common compound used in the treatment of skin infections. Hissae Yassue-Cordeiro et al. impregnated zeolite with silver sulfadiazine and tailored a zeolite–chitosan composite film. The antimicrobial activity against *S. aureus* was lacking; however, the films exhibited activity against *E. coli*, *P. aeruginosa*, and *C. albicans* [236].

Other film formulations have been utilized for the treatment of skin infections. Silver NPs are commonly used and often display promising antimicrobial efficacy. Studies incorporating silver NPs in chitosan films have shown activity against *E. coli* [237,238,239], *Bacillus sp.* [238,239], and *K. pneumonia* [239]. In addition to utilizing chitosan films as vehicles, Pansara and colleagues stabilized silver NPs with chitosan and embedded these particles in chitosan films. These films demonstrated antibacterial activity against *E. coli* in in vitro conditions as well as faster wound closure in the *E. coli*-infected wound model in rats [240].

Wang and colleagues utilized gold NPs to increase the antibacterial potential of chitosan films against *S. aureus* and *E. coli*. The modification with gold NPs significantly improved the antibacterial activity of the chitosan films against both *S. aureus* and *E. coli*. Additionally, the modified film improved wound healing in a noninfected wound model in rats, further proving the potential of these films as wound healing scaffold [241]. Additionally, other inorganic agents have been utilized to improve the antimicrobial potential of chitosan films. Hanafy et al. incorporated TiO_2_ NPs into chitosan film for the intended use in dermal wounds. These films were assessed against a range of bacteria and fungi with promising results. Both loaded films and films without NPs displayed activity against *B. cereus*, *S. aureus*, *E. coli*, *C. albicans*, and *Aspergillus niger*. In *C. albicans,* the film without NPs showed higher antimicrobial activity than the loaded films [242].

Foster and Butt found no antimicrobial activity of unloaded chitosan films compared to chitosan in solution against *S. aureus*, *S. epidermidis* and *E. coli*. Their films existed in a dry state and were unable to interact with the bacterial cells. However, the MW of the chitosan used in this study is not reported [243].

Films and membranes are often prepared in combination with other polysaccharides to improve either antimicrobial activity or mechanical properties. Archana et al. prepared scaffolds based on chitosan and pectin. These scaffolds were loaded with TiO_2_ nanorods and assessed against *S. aureus*, *E. coli*, *P. aeruginosa*, *B. subtilis*, and *A. niger*. The combination of chitosan and pectin and TiO_2_ nanorods exhibited excellent antimicrobial activity against all strains but with higher activity in bacteria [244]. Gómez Chabala et al., along with Bueno and Moraes, prepared scaffolds comprising chitosan and alginate. These scaffolds loaded with either silver NPs and aloe vera [245] or polyhexamethylene biguanide [246] both expressed antimicrobial activity against *S. aureus*; however, only the scaffold loaded with silver NPs and aloe vera displayed antimicrobial activity against *P. aeruginosa* [245,246]. Kenawy and colleagues tailored chitosan/gelatin scaffolds loaded with cinnamaldehyde and assessed their antimicrobial activity against *S. aureus*, *P. aeruginosa*, *Salmonella*, and *E. coli*. These scaffolds demonstrated antimicrobial activity in a cinnamaldehyde dose-dependent manner against all strains. The effect was generally higher against the Gram-negative strains [247]. Furthermore, chitosan has been utilized in combination with hyaluronan to produce scaffolds loaded with phosphatidylcholine dihydroquercetin. These scaffolds were challenged against *E. coli*, *K. pneumoniae*, *S. aureus*, and *Staphylococcus haemolyticus*. The antimicrobial activity of the loaded scaffolds was superior to the unloaded scaffold against all strains. The loaded scaffolds showed good anti-inflammatory and wound healing properties [248].

Many studies focus on sponge-like scaffolds and nanofibers to accommodate cell migration and wound closure. Zhou and colleagues prepared a sponge-like scaffold loaded with silver NPs and iturin, and cyclic lipopeptide and evaluated the antibacterial and antifungal effect of these formulations. In the in vivo antibacterial evaluation in *E. coli*-infected wounds in mice, the scaffold reduced the bacterial burden and demonstrated good wound healing properties. In *C. albicans*-infected wounds, the scaffolds also improved wound healing in mice [249,250]. Silver sulfadiazine is also utilized as antimicrobial compounds in these sponge-like scaffolds. Shao et al. tailored silver sulfadiazine particle scaffolds and challenged their antimicrobial potential against *E. coli*, *S. aureus*, *B. subtilis*, and *C. albicans*. The unloaded scaffolds had no antimicrobial effect on the microorganisms, but the loaded scaffolds had good antimicrobial activity in all strains in a concentration-dependent manner [251]. Dumitriu and colleagues prepared chitosan–hyaluronate sponge-like scaffolds and tailored polyelectrolyte complex between the scaffold and sulfadiazine. The complex was assessed against *Salmonella*, *Listeria monocytogenes*, and *E. coli*. Additionally, the authors evaluated the activity of LMW and MMW chitosan. The antimicrobial evaluation showed that both the LMW and MMW chitosan-based complexes had excellent antimicrobial activity against all strains; however, the LMW chitosan had better activity [252].

Hamblin et al. have extensively evaluated sponge-based bandages produced with chitosan acetate for their use in infected and burn wounds. Across different studies, this scaffold has been evaluated in vivo against *S. aureus*, *P. aeruginosa*, and *Proteus mirabilis*-infected wounds and burn injuries. It proved promising in all these wound infections and suitable as a scaffold in burn wound healing [253,254,255].

The PEO is commonly utilized together with chitosan to produce nanofibers. Chitosan–PEO nanofibers loaded with either teicoplanin [256] or vancomycin [257] have been used as scaffolds in wound healing. Here, the antibacterial potential of these fibres was assessed against *S. aureus.* Upon loading nanofibers with teicoplanin, the antimicrobial activity was significantly stronger than the free compound [256]. When loading vancomycin in the chitosan–PEO nanofibers, the same superior effect was seen against both *S. aureus* and MRSA [257].

Faccendini et al. combined chitosan with pullulan and either chondroitin sodium sulfate or hyaluronic acid and then loaded the nanofibers with montmorillonite norfloxacin nanocomposite. In the antimicrobial evaluation, the nanocomposite-loaded nanofibers exhibited antimicrobial activity against both *S. aureus* and *P. aeruginosa*; however, the activity of free norfloxacin in nanofibers was seemingly higher than when the drug was part of the nanocomposite, possibly due to slower release [258].

The antimicrobial peptide CM11 was loaded into nanofibers comprising chitosan and silk fibroin aimed at treating infected wounds. This system was evaluated against *S. aureus*, *E. coli*, *P. aeruginosa*, and resistant strains of the same bacteria. The scaffold itself did not display any antimicrobial activity; however, the nanofibers loaded with 16 µg/mL peptide exhibited inhibition against standard strains, while the effect on resistant strains was first seen at peptide concentrations of 32 µg/mL [259].

Bösiger and colleagues investigated chitosan/PEO nanofibers functionalized with the enzyme glucose oxidase. The generation of H_2_O_2_ could potentially improve microbial eradication and stimulate fibroblast proliferation. The nanofibers showed inhibition of both *S. aureus* and *E. coli* compared with the control, and this effect was observed already after two hours [260].

Monteiro and colleagues combined nanofibers with liposomes loaded with gentamicin. The authors prepared chitosan nanofibers and immobilized gentamicin-loaded liposomes on the surface of the nanofiber scaffolds. This dual system was assessed against *S. aureus*, *E. coli*, and *P. aeruginosa*. Neither the chitosan nanofibers nor the nonloaded liposomes immobilized on nanofibers displayed antimicrobial activity against any bacteria. However, the gentamicin-loaded liposomes immobilized on nanofibers exhibited a superior antimicrobial effect against all three strains [261].

## 5. Chitosan and Vaginal Infections

### 5.1. Common Vaginal Infections

Various microorganisms, including bacteria, fungi, parasites and viruses, can cause vaginal infections. Vulvovaginal candidiasis, bacterial vaginosis, and aerobic vaginitis are the most common endogenous infections. Alarmingly, in an era of AMR, the incidence of sexually transmitted infections (STIs) such as gonorrhoea, mycoplasma, chlamydia, and trichomoniasis are continuously on the rise [10].

*Candida albicans* is an innate part of the vaginal microflora; however, an imbalance in the normal flora can cause an infection. *C. albicans* is the most frequent cause of candida infections, but *C. glabrata*, *C. tropicalis*, *C. parapsilosis*, *C. crusei*, *C. stellatoidea*, and *C. lusitaniae* strains can cause vulvovaginal candidiasis as well [262]. Up to 75% of all women will experience a candida infection during their lifetime, of which 50% will experience recurrent infections despite the existing oral and topical antifungal therapies [263]. Recurrent vulvovaginal candidiasis infections are common due to persistent strains, AMR, and biofilm formation [264].

Bacterial vaginosis is one of the most widespread vaginal infections [10]. An infection originates from the overgrowth of anaerobic bacteria that are a natural part of the vaginal microflora. These anaerobic bacteria include *Bacteroides fragilis, Gardnerella vaginalis*, and *Atopobium vaginae*, of which *B. fragilis* is the most resistant strain [265]. Both oral (metronidazole and clindamycin, tinidazole and secnidazole) and topical (metronidazole and clindamycin) antibiotic treatments are currently available; however, they often fail to completely eradicate biofilms, thus, preventing successful antimicrobial therapy [8,10]. The persistent biofilm contributes to recurrent infections and might intensify the resistance to antimicrobial agents [24].

Aerobic vaginitis is caused by a dominance of *Lactobacillus* that causes abnormal vaginal microflora containing aerobic pathogens such as *Streptococcus agalactiae*, *E. faecalis*, *E. coli*, and *S. aureus* [266]. Aerobic vaginitis and bacterial vaginosis share several common features, and despite a clear difference, the two conditions often coincide in diagnosis and literature [267]. There is no standard treatment; however, because aerobic vaginitis is associated with inflammatory changes, the antibiotic clindamycin with its inherent anti-inflammatory effect is often preferred [267,268].

Trichomoniasis infection is caused by the parasite *Trichomonas vaginalis*, a flagellated protozoan that adheres to the vaginal mucosa generating colonization and persistent infection [269]. Trichomoniasis is the most common nonviral STI in women of reproductive age [270]. Oral antibiotic metronidazole is considered the first-line therapy; however, side effects, contraindication during pregnancy, and drug-resistant parasites affect the use and effectiveness of current treatment options [271].

*Neisseria gonorrhoeae* is a Gram-negative, human obligate bacteria that causes genital gonorrhoea infection [10]. Gonorrhoea is currently the second most common bacterial STI, with an estimated global incidence of 86.9 million new infections each year [272]. Intramuscular and oral single-dose antibiotics are currently first-line treatments; however, the continuous rise in AMR is threatening the effectiveness of the available therapy [273].

*Mycoplasma genitalium* is a highly prevalent STI with rapidly increasing resistance to the already limited available treatment options [274]. First-line treatment is oral antibiotic azithromycin; however, *M. genitalium* is highly prone to the development of AMR, and both *M. genitalium* and *N. gonorrhoeae* might develop into untreatable superbugs in the future [275].

*Chlamydia trachomatis* is a small obligate intracellular, Gram-negative bacterium that is the most common cause of bacterial STIs [276], with an estimated annual incidence of 127 million new infections globally [277]. Moreover, due to the asymptomatic nature of *C. trachomatis* infections, the number is believed to be considerably underestimated. The current treatment is oral antibiotics; however, AMR is expected to limit available therapy in the future [278].

The majority of vaginal infections are often present as an asymptomatic disease, implying an underestimated number of cases, increased spread, and untreated infections. The lack of treatment or unsuccessful therapy can lead to reproductive health consequences and complications in pregnancy, as well as increased risk of acquiring other STIs, including HIV [10,269,279]. A vaginal infection is often accompanied by coinfection with other sexually transmitted pathogens, especially in female populations, such as high coinfection rates by mycoplasma and chlamydia [280]. This further complicates the successful elimination of infections and contributes to the development of AMR. Hence, there is a need for new approaches, new antibiotics, and optimized delivery systems that can assure an efficient local treatment.

### 5.2. Challenges of Localized Therapy of Vaginal Infections

The vaginal environment presents several challenges for effective localized therapy. Due to the self-cleansing action of the vaginal tract, locally administered formulations often fail to persist at the site of action long enough to assure sufficient therapeutic effect [281]. Vaginal pH, the thickness of the epithelium, and the production of vaginal fluid and mucus all vary, depending on the phase of the menstrual cycle, sexual activity, age, and the presence of concomitant diseases [282].

Primarily, an adequate residence time at the site of action must be obtained to allow for therapeutic effect. The successful formulation needs to avoid rapid vaginal clearance, and in this regard, chitosan plays an important role, providing mucoadhesive properties and the possibility of prolonged residence time [70,283]. Moreover, the locally applied formulation should enable the active ingredient to overcome the vaginal mucus barrier reaching the vaginal epithelium (infection site) to provide both controlled and predictable release of the incorporated active ingredient, be uniformly distributed onto the underlying tissue, and assure a sufficiently high and maintained antimicrobial action over an adequate period within vaginal cavity [284]. In addition, the system should provide both controlled and predictable release of the incorporated active ingredient, assuring an improved therapeutic outcome. If these conditions are met, systemic exposure can be avoided, the required dose reduced, and the potential for the development of AMR can be limited [10]. Moreover, a safe treatment should be assured for pregnant patients who currently suffer from a lack of available treatment for most vaginal infections [44].

### 5.3. Antimicrobial Chitosan-Based Systems for Vaginal Application

In addition to the excellent mucoadhesive properties provided by chitosan, its intrinsic antimicrobial properties make it an attractive material in pharmaceutical applications, including those for localized vaginal therapy [74]. Another quality that chitosan can supply, of great importance in treating vaginal bacterial inflammation and infections, is the ability to disrupt bacterial biofilms [24]. The many beneficial properties enable chitosan to act as an active ingredient, carrier, mucoadhesive excipient, and an amplifier of the antimicrobial effect by working in synergy with other active ingredients [69].

Substantial research is carried out on chitosan-based applications for vaginal delivery. In the following sections, the focus is placed on vaginal formulations comprising chitosan, as active substance, carrier, excipient, hydrogel, or vaginal film, assessed regarding antimicrobial activity against common vaginal pathogens. The main findings in the discussed research are summarized in Table 3.

#### 5.3.1. Particles and Carriers

Chitosan nanoparticles (CNPs) were also tested for vaginal administration. Recent work by Facchinatto and colleagues explored CNPs containing clotrimazole, a widely used antifungal drug, for the localized treatment of vulvovaginal candidiasis [285]. Cationic N-(2-hydroxy)-propyl-3-trimethylammonium, O-palmitoyl CNPs were evaluated for anticandidal activity. The activity of clotrimazole decreased when associated with CNPs, with an increase in MIC values. However, the in vitro safety profile was improved when clotrimazole was loaded into CNPs, and the prolonged release was achieved [285].

Many natural substances show promise as alternatives to antibiotics in treating infections. Arumugam and Rajendran incorporated Callophycin A, a seaweed-derived metabolite, into CNPs and studied in vitro and in vivo anticandidal effects [286]. Callophycin A-loaded CNPs showed significant anticandidal activity both in vitro and in the vulvovaginal candidiasis animal model after six days of treatment. The CNPs alone did not exhibit antifungal activity; however, a synergetic effect between Callophycin A and CNPs was determined [286].

Murine vulvovaginal candidiasis model was also applied in a study on the anticandidal effect of thiosemicarbazide encapsulated in CNPs [287]. Thiosemicarbazide is a compound with an antifungal effect, but its biological effect is reduced due to in vivo degradation. The CNP-associated thiosemicarbazide obtained a noticeable reduction in the fungal burden after seven days of treatment, suggesting a synergistic effect [287]. CNPs alone did not affect the fungal burden.

Intravaginal application of miconazole nitrate is widely used as a treatment of vulvovaginal candidiasis. Novel delivery systems can enable an antifungal activity with lower drug concentration and dosing frequency, resulting in reduced side effects. CNPs for miconazole delivery, targeting vulvovaginal candidiasis, was studied in vivo by Amaral and colleagues [288]. The *C. albicans*-infected mice received treatment for seven days, and the antifungal effect of CNPs containing miconazole was compared to conventional miconazole cream. Both formulations showed a significant antifungal burden, and the CNPs containing a seven-fold lower drug concentration than the cream obtained a similar therapeutic effect [288].

The combination of available antifungal compounds and natural substances can enable an enhanced activity of existing drugs. Farnesol is found in plant extracts and has shown effectiveness against several microbials, including the antifungal effect. CNPs containing miconazole or farnesol have been assessed for their anticandidal activity to explore the possible adjuvant effect of farnesol [289]. Both in vitro and murine vulvovaginal candidiasis models were employed. The in vitro susceptibility test weakened the theory of the synergistic effect of miconazole and farnesol, with no enhanced effect by including farnesol in the formulation. In vivo results showed that the combination of miconazole and farnesol in CNPs was the most effective treatment; however, not significantly better compared to miconazole CNPs [289].

Electrospraying as a production technique to generate CMPs and CNPs is gaining attention. Moreno et al. developed chitosan microcapsules containing dry extracts of Argentinean medicinal plants through electrospraying, targeting the localized treatment of vulvovaginal candidiasis [290]. Argentinean medicinal plants have been shown to possess biological properties. These properties were maintained when encapsulated in chitosan microcapsules, and a strong antifungal capacity was obtained in the in vitro *C. albicans* challenge [290].

Doxycycline is a widely used antibiotic for various vaginal infections, including bacterial vaginosis, chlamydia and mycoplasma. Cover et al. studied the antibacterial activity of doxycycline-loaded CNPs and explored the synergy of drug and polymer [291]. CNPs containing doxycycline expressed a significant reduction in the viability of *E. coli* in vitro. The CNPs alone did not inhibit the bacterial growth; however, the cytotoxicity related to free doxycycline was significantly reduced when entrapped in CNPs, suggesting a prominent role of chitosan in improving the biocompatibility of drugs [291].

Microscale chitosan particles have also been assessed as carriers with the potential in localized treatment of vaginal infections. Cirri et al. developed chitosan and chitosan-alginate microspheres containing metronidazole for vaginal administration [265]. Antibiotic metronidazole is commonly used to treat bacterial vaginosis, generally administered orally, causing several side effects. Hence, the appropriate formulation for local administration could improve the therapy considerably. Chitosan–alginate microspheres expressed superior growth inhibition of *B. fragilis* in vitro compared with chitosan microspheres [265].

The same group previously evaluated the antibacterial effect of chitosan–alginate microspheres containing antibiotic cefixime, used in the treatment of various infections, including aerobic vaginitis and gonorrhoea. Cefixime microspheres had the ability to reduce the *E. coli* viability to the same extent as a free drug, suggesting improved effect due to the improved mucoadhesive ability of the system [292].

In recent years, also fungal chitosan has gained increased attention. Chitosan with beneficial properties for healthcare applications can be obtained from fungi, as an alternative to the commonly used marine source [21]. Elmi et al. developed CNPs with chitosan obtained from *Penicillium* fungi and evaluated their in vitro antimicrobial effect against *T. vaginalis* [293]. Compared with the antimicrobial drug metronidazole, applied as positive control, the effectiveness of CNPs was dependent on the polymer concentration and duration of the exposure. Nevertheless, CNPs significantly eliminated the *T. vaginalis* burden in vitro [293].

*N. gonorrhoeae* is the second most common bacterial STI, and the available treatment options are continuously reduced due to the AMR development [10]. Alqahtani et al. formulated CNPs and evaluated their antibacterial effect against various *N. gonorrhoeae* strains in vitro, including strains sensitive to conventional antibiotics and multidrug-resistant strains [294]. The developed CNPs expressed antibacterial effect against all tested strains, confirming the therapeutic potential of CNPs also for high-level resistant *N. gonorrhoeae* [294].

#### 5.3.2. Coating Material and Excipients

The application of chitosan as a component in vaginal drug delivery systems is versatile and entails many opportunities and advantages [75]. Surface modification of NPs can provide the necessary properties to improve topical therapy, and the use of chitosan as a coating material is a widely known approach for generating mucoadhesive NPs suitable for vaginal formulations. Calvo et al. designed chitosan-coated nanocapsules as carriers for azole antifungals tioconazole and econazole [295]. Chitosan-coated nanocapsules maintained the antifungal activity against *C. albicans* for both drugs in vitro. Unloaded nanocapsules and chitosan on their own did not express anticandidal activity [295]. In another study, the surface of PLGA NPs was modified with chitosan to obtain mucoadhesive properties [296]. Clotrimazole was loaded into the PLGA NPs and challenged against *C. albicans*, comparing the activity of chitosan-coated and noncoated NPs containing drug to the free drug. Chitosan-coated PLGA NPs showed superior anticandidal activity in vitro and increased the effectiveness of clotrimazole [296].

Andersen et al. developed a chitosan-based delivery system with chitosan both entrapped in liposomes and available on the liposomal surface as a coating layer [27]. The chitosan-based formulation containing metronidazole and the drug-free formulation were challenged against *C. albicans* in vitro. A superior antifungal activity was observed for the chitosan formulations compared to free drug, plain liposomes, and Carbopol-containing liposomes. Moreover, the inhibition of *C. albicans* was equal for the chitosan-based delivery system containing metronidazole and the drug-free formulation, confirming the intrinsic antifungal properties of chitosan [27].

Recently, microemulsion associated with chitosan was also assessed for its potential in the topical treatment of vulvovaginal candidiasis [297]. Both chitosan and herbal medicine, *Stryphnodendron adstringens* extract, *Melaleuca alternifolia* essential oil and tea tree oil, was incorporated in the microemulsion, replacing the aqueous phase. All constituents of the microemulsion expressed *C. albicans* activity in vitro; however, compounds were tested separately, and the effect of formulation needs to be further evaluated to confirm the favourable influence of the chitosan delivery system in vulvovaginal candidiasis treatment [297].

Metronidazole is primarily applied in treating bacterial vaginosis; however, it often fails to eradicate possible coinfection by *Candida* spp. The combination of metronidazole and chitosan can obtain an effective treatment of such coinfection due to the inherent antifungal properties of chitosan. Perinelli et al. prepared metronidazole-associated chitosan, either as free polymer or CNPs, added to hydroxypropylmethyl cellulose hydrogel [298]. Anticandidal activity in vitro was assessed and compared to free metronidazole in solution or hydrogel, and the antimicrobial activity was increased in the presence of chitosan. However, the presence of the drug in formulations containing chitosan as free polymer or as CNPs did not increase the antimicrobial activity against tested *Candida* spp. strains. Thus, proving that metronidazole did not influence the intrinsic antifungal effect of chitosan [298].

In another study, chitosan-based microplatelets was developed for the intravaginal delivery of amphotericin B deoxycholate, aiming at localized therapy of vulvovaginal candidiasis [299]. The chitosan microplatelets containing amphotericin B were dispersed in Pluronic^®^ F127 hydrogel, and the antifungal activity against *C. albicans* was assessed both in vitro and in vivo. The optimized formulation obtained a complete infection cure in the murine vulvovaginal candidiasis model. The in vitro evaluation corroborated these findings. Moreover, the chitosan-based microplatelets exhibited synergistic activity with associated amphotericin B in the in vitro antifungal evaluation [299].

Chitosan can interact with other polymers, forming complexes with beneficial properties. Darwesh et al. evaluated the anticandidal effect of chitosan and anion–polyelectrolyte complex vaginal inserts for the localized delivery of antifungal drug fluconazole [300]. The antifungal effect was assessed both in vitro and in the vulvovaginal candidiasis animal model. Fluconazole vaginal insert showed improved antifungal activity both in vitro and in vivo compared with the free drug [300].

Natural origin substances, as alternatives to existing antimicrobials, strengthen the potential of improved and successful therapy of complicated and recurrent infections. Natural molecule curcumin has shown to possess various beneficial properties, including anti-inflammatory and antifungal. Salmazi et al. aimed to exploit these properties by loading curcumin into liquid crystal precursor mucoadhesive system containing chitosan targeting vulvovaginal candidiasis [301]. While the delivery system did not inhibit *C. albicans* growth on its own, the curcumin-loaded liquid crystalline formulations expressed anti-candida activity in vitro. Moreover, the antifungal effect was considerably more potent when curcumin was associated with formulation than free curcumin [301]. Subsequently, a similar system of liquid crystalline containing chitosan for the vaginal delivery of curcumin was assessed for its antifungal activity in vivo and in vitro, as well as on *C. albicans* biofilm [302]. The optimized formulation was shown to improve the antifungal potency of curcumin in vitro, and activity was seen when challenged against fluconazole-resistant strains. The fungal burden in the murine vulvovaginal candidiasis model was significantly decreased by the formulation after four days of treatment, corroborating the in vitro findings. Moreover, the optimized formulations efficiently reduced the in vitro growth of biofilm, obtained using clinical strains [302].

The use of chitosan as an excipient in vaginal tablets can provide the mucoadhesive properties necessary to achieve successful localized therapy. Fitaihi et al. developed chitosan-based vaginal tablets containing fluconazole for vulvovaginal candidiasis treatment [303]. The tablet formulation increased the antifungal activity of fluconazole against *C. albicans* with a greater inhibition zone compared with the free drug [303]. Chitosan-based vaginal tablets have also been explored as a delivery system for the natural active ingredient, namely *Chelidonii herba* lyophilized extract [304]. The chitosan-based tablets containing extract were challenged against various *Staphylococcus* spp., *P. aeruginosa*, and *C. albicans* to evaluate their in vitro antimicrobial activity. However, the tablet formulation detained the activity of *Chelidonii herba* extract, and a superior effect was obtained by the free extract [304].

A novel approach for improved treatment of vaginal candidiasis is the in situ vaginal gel containing gel flakes. Gel flakes have a thin threadlike shape with polygonal structures. Abd Ellah et al. developed gel flakes containing antifungal drug ketoconazole via gelation of gellan gum solution containing drug mixture (ketoconazole /β-cyclodextrin) in the presence of chitosan, forming the chitosan and gellan gum gel flakes [305]. The gel flakes containing ketoconazole were further incorporated into in situ thermosensitive gel, and the formulation was assessed for in vitro and in vivo antifungal activity. In vitro anti-candida activity of the formulation was compared to free ketoconazole and marketed terconazole vaginal cream, and the inhibition zone after 48 h was significantly better for the gel flakes formulation. In a pilot study involving 100 vulvovaginal candidiasis patients, the efficacy of ketoconazole gel flakes formulation was equivalent to the marketed product, confirming the in vivo effect of the novel system [305]. A similar approach was developed by Permana et al. comprising thermosensitive in situ vaginal gel combined with the gel-flake system of itraconazole-containing solid dispersion powder [306]. In vivo antifungal activity was evaluated using a vulvovaginal candidiasis animal model. It was found that the combination of the solid dispersion, gel-flakes, and in situ gel significantly improved the anticandidal effect of itraconazole [306].

Vaginal inserts based on chitosan complexes have been studied for the delivery of other antibiotics as well. Chitosan and alginate complex for vaginal delivery of chlorhexidine was evaluated for its antimicrobial activity towards both *C. albicans* and *E. coli* [307]. Vaginal inserts increased the antimicrobial activity of chlorhexidine in vitro after 24 h of incubation [307]. Subsequent studies by the same group explored the antimicrobial activity of chlorhexidine in a similar delivery system. Vaginal inserts based on chitosan and carboxymethylcellulose complexes containing chlorhexidine was challenged against the same pathogens [308]. Results aligned with earlier findings suggesting the future applicability of vaginal inserts against vaginal infections, such as candidiasis and aerobic vaginitis.

*Staphylococcus* spp. is naturally found on mucosal surfaces, and to explore the antibacterial effect of chitosan, Jøraholmen et al. challenged chitosan-coated liposomes against methicillin-resistant or sensitive strains of *S. aureus* and *S. epidermidis* [8]. The activity of chitosan-based formulations (free of drug) was compared to noncoated liposomes (free of chitosan) and antibiotic vancomycin. The chitosan-coated liposomes expressed an antibacterial effect against *S. epidermidis* in all tested concentrations, while merely the highest chitosan concentration showed activity against *S. aureus*. Noncoated liposomes did not show any antibacterial impact, confirming the antimicrobial properties of chitosan [8].

In another study targeting bacterial vaginosis, Perioli et al. prepared mucoadhesive vaginal tablets containing chitosan, as well as antibiotic metronidazole, and tested their ability to inhibit *B. fragilis* growth in vitro [309]. Tablets with various ratios of chitosan and other polymers were assessed, and superior antibacterial inhibition was seen in the 1:1 chitosan and PVP tablets [309].

Metronidazole vaginal tablets comprising chitosan were also developed by Lupo and colleagues [310]. Synthesized S-protected chitosan was added as a mucoadhesive excipient, and to assess the potential in localized treatment of aerobic vaginitis, their ability to inhibit *E. coli* in vitro was measured. Vaginal tablets with S-protected chitosan exhibited antimicrobial effect; however, with a reduced growth inhibition zone compared to that of free metronidazole and metronidazole released from unmodified chitosan, possibly due to the prolonged release from the vaginal tablets [310].

Another study on the antimicrobial effect of chitosan, excluding the influence of the antibiotic drug, was performed by Pradines et al., who produced chitosan-coated poly(isobutyl cyanoacrylate) (PIBCA) NPs [271]. Their findings suggested that the in vitro antimicrobial effect against *T. vaginalis* was related to the PIBCA NPs, however, merely when the NPs were chitosan-coated [271]. The same group evaluated the activity against *T. vaginalis* using the same chitosan-coated NPs containing the antibiotic drug metronidazole [270]. Chitosan as a pharmaceutically active ingredient was confirmed, and its activity might be due to morphological changes of *T. vaginalis* caused by the chitosan-coated NPs [270]. In another study, metronidazole-associated chitosan was added to a thermosensitive Pluronic^®^ F127 hydrogel aiming for topical therapy of vaginal trichomoniasis infection [311]. The effect of free drug metronidazole in the formulation, as well as drug-free formulation, were assessed for their antimicrobial activity against *T. vaginalis* in vitro. The antimicrobial effect of metronidazole was maintained in hydrogel formulation, in contrast to the action of metronidazole in solution. Drug-free hydrogel decreased the *T. vaginalis* viability as well; however, high concentrations of chitosan was required, confirming the intrinsic antimicrobial activity of chitosan [311]. *T. vaginalis* motility in biological fluids is an important quality in the infectivity of the parasites. Subsequent studies aimed to evaluate the effect on *T. vaginalis* motility [312]. A similar thermosensitive hydrogel containing chitosan was used, except no drug was added to the formulation. The hydrogel containing chitosan was able to reduce *T. vaginalis* motility in biological fluids; however, Pluronic^®^ F127 hydrogel free of chitosan more efficiently immobilized the parasites, enabling prevention of infection [312].

#### 5.3.3. Polymer-Based Vaginal Gels

Semisolid formulations in the form of vaginal gels are one of the most extensively investigated vaginal dosage forms [282]. In particular, polymer-based hydrogels are known as a promising strategy to achieve improved localized therapy of vaginal infections [262]. Numerous studies have been conducted evaluating chitosan hydrogel as an excipient in pharmaceutical formulations, a carrier of pharmacological substances, a secondary vehicle for particulate delivery systems, and an intrinsic antimicrobial aiming for the localized therapy of vaginal infections [74].

Chitosan hydrogel is proven to own intrinsic antimicrobial properties. The potential of chitosan hydrogel as treatment of vulvovaginal candidiasis was evaluated, and drug-free hydrogel was challenged against a wide range of *Candida* spp. in vitro. Results demonstrated the varying potency against the different *Candida* strains; however, they confirmed the intrinsic antifungal properties of chitosan hydrogel [313]. The antimicrobial action was suggested to be caused by membrane damage due to the interaction between the protonated amino groups of chitosan and the negatively charged membrane proteins of *Candida* spp. This theory is supported for other formulations of chitosan as well [26]. The choice of active ingredient can influence the antimicrobial activity of chitosan hydrogel, and Palmeira-de-Oliveira et al. tested the possible synergistic effect of *Thymbra capitata* essential oil and chitosan [314]. In vitro antifungal evaluation confirmed an increased activity by chitosan hydrogel associated with *Thymbra capitata* essential oil. Moreover, the formulation was able to disrupt the *Candida* biofilm in a dose-dependent manner [314]. In another study, methanolic extract of *Mitracarpus frigidus* was incorporated into chitosan hydrogel [315]. The murine vulvovaginal candidiasis model was used to evaluate the in vivo antifungal activity, and the activity of chitosan hydrogel containing extract was compared to the marketed product of antifungal clotrimazole cream. The *Mitracarpus frigidus* chitosan hydrogel expressed a superior antifungal effect after three days of treatment and an equal impact after six days of treatment, compared to the marketed product [315].

The antimicrobial properties of chitosan hydrogels can be influenced by the MW, DDA, ionic strength, and pH [74]. Şenyiğit et al. studied chitosan hydrogels of LMW, MMW, and HMW containing the antibiotic drug, miconazole, or econazole, for their in vitro antifungal effect [316]. *C. albicans* inhibition was superior; nevertheless, authors suggested MMW hydrogel as superior for the topical therapy of vulvovaginal candidiasis due to favourable mucoadhesive, mechanical, and release properties. Unexpectedly, chitosan hydrogel on its own did not express any inhibition of fungal growth [316].

The use of nanosystems can increase the potency of incorporated active ingredients, both conventional antibiotics and natural substances, and in combination with mucoadhesive chitosan hydrogel, the localized treatment of vaginal infections can be improved. Dos Santos et al. combined the nanoemulsion containing *Pelargonium graveolens* essential oil with chitosan hydrogel aiming to optimize the activity for the successful treatment of vulvovaginal candidiasis [317]. Nanoemulsions, with and without essential oil, and chitosan hydrogel with and without the nanoemulsions containing essential oil were challenged against various *Candida* spp. in vitro. The chitosan hydrogel on its own expressed antifungal activity against several strains and was found superior to the nanoemulsion containing essential oil for most strains. Moreover, the chitosan hydrogel-thickened nanoemulsion containing *P. graveolens* essential oil showed a significant reduction in MIC compared to plain essential oil [317].

Persistent bacterial biofilms might be a contributing factor to recurrent infections and the failure to completely eradicate both vulvovaginal candidiasis and bacterial vaginosis. Silva-Dias et al. used a mouse subcutaneous foreign body system to evaluate the effect of chitosan hydrogel on C*andida* biofilm [318]. The efficacy of chitosan was confirmed by significantly reduced biofilm formations both in vitro and in vivo and its ability to disrupt a preformed biofilm [318]. In another study, iminoboronate-chitosan hydrogels were prepared using 2-formylphenylboronic acid as crosslinking agent [319]. Hydrogels were evaluated regarding antibiofilm and fungicidal activity. Iminoboronate-chitosan hydrogels inhibited the formation of *Candida* biofilms in vitro and reduced their metabolic activity by more than 99.5%. Moreover, an efficient fungicidal activity in biomimetic conditions was obtained [319].

The ability of chitosan hydrogel to act on biofilm was confirmed against bacterial vaginosis-associated biofilm as well. Kandimalla et al. found that low concentration chitosan hydrogel effectively eradicated *P. aeruginosa* biofilms in vitro [24].

The chitosan-based hydrogel can also be used as a secondary vehicle, incorporating nanocarriers. Jøraholmen et al. developed liposomes-in-hydrogel to evaluate the intrinsic antibacterial properties of chitosan by assessing the in vitro activity against *Staphylococcus* spp. [8]. Various concentrations of chitosan hydrogel containing drug-free liposomes were tested against methicillin-resistant or sensitive strains of *S. aureus* and *S. epidermidis*. Antibacterial activity against all tested strains was demonstrated for all chitosan concentrations. In this study, the effect of formulation on the antibacterial properties of chitosan was assessed. As mentioned in the section on excipients, chitosan-coated liposomes inhibited the growth of *S. epidermidis* in all concentrations; however, *S. aureus* was merely inhibited by the higher chitosan concentration (0.3%). These findings suggest the superiority of chitosan formulated as hydrogel and that the antibacterial effect of chitosan is dependent on bacteria as well as the type of formulation [8].

In a recent study, vaginal rings were manufactured using 3D printing [320]. The vaginal rings were filled with polymer-based gels containing antibiotic metronidazole, and their effect was evaluated in vitro against *C. albicans* and *E. coli*. The empty vaginal ring, metronidazole-containing vaginal ring, chitosan and hydroxyethyl cellulose-containing vaginal ring, and chitosan and metronidazole-containing vaginal ring were included in microbiological evaluation over 24 h. The effect against *C. albicans* was initially superior for the chitosan and metronidazole-containing vaginal ring; however, after eight hours, the fungal growth was similar to the untreated controls. In the *E. coli* challenge, all preparations expressed an antibacterial effect. The vaginal ring containing chitosan and metronidazole obtained a bactericidal effect after 24 h and was found superior. The findings confirmed a synergistic effect of chitosan and metronidazole against *E. coli* [320].

The antirheumatic drug, auranofin, exhibits significant trichomonacidal activity in low concentrations both in vitro and in vivo in a murine infection model [327]. To avoid simultaneous adverse effects and exploit the beneficial properties of auranofin in topical therapy of trichomoniasis, Zhang et al. incorporated the drug into PLGA NPs [321]. NPs were subsequently incorporated into a β-glycerophosphate and chitosan-based thermosensitive hydrogel and challenged against *T. vaginalis* in vitro. Although the auranofin NPs in the hydrogel formulation was less potent than the free drug, the formulation managed to completely inhibit parasite growth in a dose-dependent manner. Drug-free formulation did not influence the *T. vaginalis* growth [321].

The combination of two delivery systems, a drug nanocarrier incorporated into a chitosan hydrogel, has recently been evaluated for its antibacterial effect, namely the liposome-in-hydrogel delivery system. Jøraholmen et al. aimed to prove the in vitro antichlamydial effect of natural substance resveratrol in a suitable delivery system [276]. *C. trachomatis* infected cells were exposed to free resveratrol, liposomal resveratrol, resveratrol liposomes-in-hydrogel and respective controls free of resveratrol. All resveratrol formulations reduced the number of infected cells, and compared with free resveratrol, a superior antichlamydial activity was seen for resveratrol liposomes-in-hydrogel in the lower concentrations. The intracellular bacteria were not affected by the chitosan-based hydrogel free of resveratrol [276].

#### 5.3.4. Vaginal Films

Vaginal films are considered solid dosage forms and are usually thin, soft, and flexible and disperse or dissolve in contact with vaginal fluids. Films are formulated using polymers, either one or a combination of polymers, and due to its mucoadhesive and antimicrobial properties, chitosan is highly suitable for vaginal film preparation [282]. Mishra et al. prepared a mucoadhesive vaginal film containing fluconazole by using polymers chitosan and pectin with glycerol as plasticizer [322]. To assess the antifungal activity, the optimized vaginal film was challenged against *C. albicans* in vitro. Moreover, the *Lactobacillus* inhibition was studied to exclude adverse effects on vaginal flora. Compared with a marketed gel containing fluconazole, the chitosan-based vaginal film obtained a similar *C. albicans* growth inhibition. Moreover, the optimized vaginal film did not suppress the growth of *Lactobacillus,* and no significant difference was seen in growth inhibition compared to the marketed product [322].

Tioconazole has been shown to hold higher activity against *C. albicans* compared with some of the commonly applied azole antifungals. Calvo et al. prepared vaginal chitosan-based films containing tioconazole, with chitosan and a combination of chitosan and hydroxypropyl methylcellulose [323]. The in vitro biological activity of drug-free films and films loaded with tioconazole was evaluated over time against *C. albicans* and compared to tioconazole vaginal capsules. A superior and more rapid anticandidal effect was observed for the vaginal films than both capsules and free drug in the 96-h assay. The inhibition zone for the tioconazole vaginal capsules was reduced by 89% after 96 h. Two hours into the study, a similar reduction for the vaginal films was 24–41%, indicating the beneficial sustained drug release from the beneficial sustained drug release films. The highest *C. albicans* inhibition was seen for the vaginal film developed with chitosan as the only polymer loaded with tioconazole. Moreover, drug-free chitosan vaginal film presented fungicidal activity [323].

In a different approach, an econazole vaginal delivery system comprising chitosan and poloxamer matrix containing drug-loaded Eudragit MPs was developed [324]. The in vitro antifungal activity of chitosan-based matrices with econazole MPs was compared to the activity of matrix with unloaded MPs and merely matrix. All matrices containing econazole produced an equally sized *C. albicans* inhibition zone, while no inhibition was seen for the drug-free formulations [324].

Tentor et al. developed the chitosan-based membrane for vaginal application as a potential formulation for the management of bacterial vaginosis [325]. The membrane was prepared by allowing the dried alginate hydrogel containing metronidazole to swell in chitosan solution. Chitosan penetrates the alginate dry film, forming the membrane. The in vitro antimicrobial activity of drug-loaded membranes was evaluated against *S. aureus* and *G. vaginalis* and compared to the action of drug-free membrane and free metronidazole. The chitosan-based membrane did not restrain the antimicrobial effect of metronidazole and expressed equal activity against both strains compared to the free drug [325].

In another study, chitosan on its own and chitosan and poly(2-ethyl-2-oxazoline) was used to develop vaginal films [326]. The in vitro antibacterial activity was evaluated against *E. coli* and *S. aureus,* comparing the effect of drug-free films and films loaded with ciprofloxacin. The drug-free films did not express antibacterial activity against *S. aureus*; however, the film with a combination of polymers expressed some activity against *E. coli*. Moreover, the vaginal films containing ciprofloxacin enhanced the action of the drug, with superior growth inhibition against both bacteria compared with the free drug [326].

## 6. Other Localized Antimicrobial Therapies

### 6.1. Ocular Infections

Ocular bacterial infections, including keratitis, conjunctivitis, blepharitis, endophthalmitis, dacryocystitis, and orbital cellulitis, are exciting targets to explore the extended role of chitosan as an active pharmaceutical ingredient. Common causes of infections comprise *S. aureus*, coagulase-negative *Staphylococcus*, *P. aeruginosa*, *Streptococcus pneumonia*, *E. coli*, and *Serratia* species [328]. However, successful localized therapy is highly dependent on the performance of the drug vehicle–delivery system. It is well established that the ocular site represents one of the most challenging mucosal sites; the corneal penetration is extremely limited (up to 5%) due to eye reflection, blinking, and tear fluid turnover. In addition, a very limited volume of the formulation can be instilled to the eye [329,330]. The cornea, the anterior eye region of the eye, is comprised of the epithelium, stroma, and endothelium. It serves as a mechanical barrier for the drug delivery systems. Due to the cornea´s superficial layers, the lipophilic epithelium, and the hydrophilic stroma, the permeation of different active ingredients is highly reduced [331]. The presence of high lipid content in the epithelium and endothelium limits the passage of hydrophilic molecules [332]. Rapid elimination, poor bioavailability, on-site irritations, accompanied by low patients’ compliance, limit the success of localized ocular delivery [333]. To assure a sufficient residence time at the site of infection, the mucoadhesiveness combined with intrinsic antimicrobial activity make chitosan highly relevant for the treatment of ocular infections.

Chitosan and its derivatives have been widely studied as a building block for various advanced drug delivery systems destined for ocular administration [333]. Moreover, chemical modifications have been introduced to enhance its mucoadhesiveness. Among ocular infections, widely prevalent bacterial conjunctivitis affecting patients of all ages is one of the common targets. Clarithromycin-loaded CNPs were shown to be effective in managing bacterial conjunctivitis due to increased precorneal residence time [334].

Chitosan was often studied in combination with other pharmaceutical excipients; for example, chitosan/cyclodextrin nanospheres were evaluated in vitro for their potential to improve the delivery of levofloxacin, a third-generation tricyclic quinolone. Novel NPs exhibited 2-fold increased antibacterial activity against both Gram-positive and Gram-negative bacteria compared with the free drug [328]. Sustained release of levofloxacin was also achieved utilizing thermosensitive chitosan-based hydrogel targeting postoperative endophthalmitis. The aim was to assure the drug´s release for at least seven days after a single instillation post cataract surgery [29].

Mirzaeei and colleagues proposed nanofibers comprising PVA/chitosan single-layered as well as PVA/chitosan/Eudragit RL100 multilayered electrospun nanofibers as a superior vehicle for ocular delivery of antibiotic ofloxacin. Animal studies revealed that nanofibers effectively retained the drug concentration in the tear fluid of rabbits above the MIC90% for up to 95 h [335].

Infectious keratitis is characterized by a concurrent infection and inflammation of the cornea that, if untreated, can cause the destruction of the cornea. Bacterial keratitis is mainly caused by *P. aeruginosa* and *S. aureus*; however, the infection can also be caused by fungi, parasites, or viruses. CNPs were evaluated as a superior delivery system for tobramycin sulphate in vitro and ex vivo conditions and showed improved performance [336].

Novel delivery systems offer the possibility to simultaneously deliver anti-infective and anti-inflammatory drugs, such as chitosan and chitosan derivates-based nanocarriers bearing dexamethasone sodium phosphate and chloramphenicol proposed by Karava et al. [330]. The derivatives improved both the mucoadhesive properties and antimicrobial activity against *S. aureus* and *E. coli*. Gade et al. prepared a drug-eluting polymeric contact lens for the effective delivery of moxifloxacin and dexamethasone. The drug-loaded contact lens exhibited significantly greater corneal drug distribution and superior in vitro and in vivo antimicrobial activity compared with the standard formulation, suggesting broader applicability in the prevention of postoperative ocular infections [329].

Fungal keratitis (keratomycosis) is an ocular infection comprising corneal inflammation [337]. It is caused by filamentous fungi such as *Aspergillus flavus*, *A. fumigatus* and *Candida*. Its incidence and frequency are alarmingly increasing due to the increased use of contact lenses and the number of immunocompromised patients [331,333]. Luliconazole, an imidazole antifungal drug, was successfully incorporated in chitosan-based NPs with superior residence time and improved ocular tolerance as a novel formulation to treat fungal keratitis [333]. Voriconazole, a second-generation antifungal, was incorporated in mucoadhesive (chitosan-coated) cubosome and challenged in a rabbit model. The novel chitosan-coated cubosomes significantly improved transcorneal permeation and ocular residence [331].

Verma and colleagues developed trimethyl chitosan-coated niosomes for natamycin, with improved mucoadhesive properties, drug release profile, and limited irritancy. Trimethyl chitosan improved the paracellular permeability of the epithelial barrier present at the ocular surface. The novel system exhibited improved natamycin PK/PD in the rabbit model [337].

### 6.2. Buccal Treatment

The oral cavity has been highlighted as a site for bioadhesive drug delivery for a long time [338]. It is highly suitable for local drug delivery into the oral cavity targeting toothaches, fungal infections, gingivitis, mucosal lesions, and periodontitis. Those conditions are particularly challenging to treat among pediatric patients [339]. Utilizing mucoadhesiveness of chitosan to prolong formulations´ residence within the buccal cavity has been explored for a variety of chitosan-based delivery systems.

Among commonly studied delivery systems, mucoadhesive buccal films gained attention due to their superior palatability. The antifungal multilayer system composed of cationic chitosan and anionic pectin was developed for controlled delivery of clotrimazole by Potas et al. [340]. Chitosan improved the antifungal activity of the drug, indicating the system´s potential in the treatment of resistant oral candidiasis.

The monolayer and bilayered mucoadhesive film and wafer formulations comprising chitosan and hydroxypropyl methylcellulose (HPMC) were developed by Timur et al. to deliver cefuroxime axetil [341]. Chitosan enhanced the antimicrobial activity, while its penetration enhancing property assisted a penetration of the drug into deeper layers of the oral tissues. Abou Hussein and colleagues studied chitosan blended with PVA-based buccal film as a vehicle for cetylpyridinium chloride, a quaternary pyridinium antiseptic, confirming its superior antimicrobial activity and good kill-time against *Streptococcus* mutants [339].

A clear and relatively unexplored potential of chitosan as an intrinsic antimicrobial has been recently confirmed by Li et al., who showed the inherent antibacterial activity of chitosan scaffolds alone for the first time [342]. The noncrosslinked chitosan scaffold was challenged against typical oral pathogens, Gram-negative *Porphyromonas gingivalis*, and Gram-positive *S. mutans*. Both pathogens were killed at six hours. Interestingly, β-glycerophosphate crosslinked scaffolds showed no antibacterial activity. The findings suggest that the noncrosslinked chitosan scaffolds offer both antimicrobials as well as wound healing potential. Considering that they are drug-free, their suitability in localized antimicrobial therapy is even more important [342].

Oropharyngeal candidiasis is one of the most prevalent fungal infections [343]. The authors prepared the polyelectrolyte films of chitosan-gum-arabic and chitosan-polygalacturonic acid loaded with nystatin. Both formulations exhibited significant antifungal activity. Interestingly, a minor effect was seen in the placebo formulation comprising only chitosan and gum arabic. Still, no antimicrobial effect was found in the placebo (drug-free) films containing polygalacturonic acid. Polymeric films comprising chitosan, carbopol, gelatin, gum arabic, and alginate were used to prepare the polymeric matrices for miconazole nitrate. The antifungal challenge against the five most important fungal opportunistic pathogens of the *Candida* genus revealed that the chitosan-gelatin and chitosan-carbopol matrices exhibited superior in vitro activity [344]. Interestingly, chitosan alone showed antifungal activity. The mucoadhesive sponges containing a mixture of tenoxicam and miconazole nitrate were prepared to target recurrent aphthous ulcer, an inflammatory disease accompanied by a candida infection. Medicated chitosan (2%) sponges caused a significant decrease in the acetic acid-induced ulcer size in rats after six days of treatment compared with negative and positive controls. Additionally, histopathological examination showed faster healing with complete restoration of the normal oral histology in rats [345].

Recently, awareness has been raised regarding the formation of biofilms on dental and mucosal surfaces. Effective eradication of biofilms with antimicrobials requires both a high concentration and long exposure time. The miniaturized drug delivery devices, known as microcontainers, able to deliver the antimicrobial peptide nisin to an oral multi-species biofilm were proposed by Birk et al. [346]. The antibacterial effects of novel microcontainers exhibited a faster onset (after three hours) than solution-based delivery (after nine hours).

Mucositis, an oral inflammation with painful erythematous lesions, is a serious condition accompanying chemotherapy. Efficient localized treatment with mucosal protectors, keratinizing agents, and local antimicrobials could be achieved by combining a thermoreversible and mucoadhesive spray hydrogel such as poloxamer–chitosan mixtures. Caricato et al. developed a spray thermogel for oral delivery of an active natural onion extract. The onion extract antimicrobial and antioxidant activities, as well as the high biocompatibility of novel formulation, should be further confirmed in in vivo conditions [347].

Chitosan could be further exploited in different fields of dentistry. From its remineralizing property assisting hardening of tooth tissues, improved healing of post extraction oral wounds, a reduction in bacterial biofilm when applied as dental cements, to widely explored antibacterial, antifungal, and hemostatic properties. Ciciu et al. provided an overview of the clinical studies on chitosan-based formulations for dental use [348]. The role of chitosan in treating various oral health conditions with emphasis on periodontitis and endodontic infections has been comprehensively summarized by Fakhri et al. [349]. Periodontitis and dental caries are two of the most prevalent dental bacteria-caused diseases. Their potential link to cardiovascular diseases, diabetes, and central nervous system disorders, brought focus to the urgent need to treat the infections early and efficiently [342]. *P. gingivalis* is considered as the primary pathogen causing periodontitis, prone to biofilm formation [350]. In addition, *S. mutans* is considered the main causative pathogen for dental caries [351].

Since our review is focused on the delivery systems, we enlist herewith some of the interesting delivery systems studied for the improvement of the antimicrobial activity of chitosan. For example, CNPs reduced the *S. mutans* adherence up to 93.4% [352]. Covarrubias et al. developed a hybrid copper–chitosan NPs with antibacterial activity against cariogenic *S. mutans* and confirmed that chitosan interacts electrostatically with hydroxyapatite and the bacterial cell wall enhancing the antibiofilm activity of copper–chitosan NPs [353].

Antibiofilm activity of CNPs against *S. mutans*, *E. faecalis*, and *C. albicans* multi-species infections was confirmed by Elshinawy and colleagues [354]. The action against *E. faecalis* contaminated root canals was also reported by del Carpio-Perochena et al. [355].

Highly relevant is the impact of the chitosan coating of medical devices on reducing the contamination possibility. Namangkalakul et al. reported that HMW chitosan incorporated into carboxymethylcellulose is a superior antifungal denture adhesive. Moreover, the authors suggested that the MW affects the antifungal activity; the LMW chitosan failed to reduce the fungal load of acrylic resin discs [356]. The issue remains debatable. Various chitosan-based delivery systems have been evaluated for the treatment of periodontitis, such as microspheres berrying clindamycin, tetracycline, doxycycline, and chlorhexidine. For more detailed descriptions, the readers are referred to Fakhri et al. [349]. The same review summarizes the various chitosan-based NPs, gels, and fibres targeting the same dental inflammation linked infections.

A more detailed description of the preparation methods applied in the manufacturing of novel chitosan-based delivery systems for dental use is offered by Zhang and co-authors [357].

### 6.3. Nasal Route

Chitosan has been primarily used in nasal drug delivery as a pharmaceutical excipient, assuring required bioadhesion of the system. However, its role as antimicrobial needs to be expanded. For example, excessive use of anti-influenza drugs is believed to contribute to the emergence of drug-resistant viruses. Therefore, it is important to discover new treatment approaches [7]. In a pioneering work, Su et al. found that at high concentrations, the HMW chitosan could inactivate murine norovirus MNV-1 [358]. Li and colleagues suggested that carbohydrate-functionalized chitosan fibre could be utilized for influenza virus capture [359]. Zheng and colleagues reported that the innate immune response could be stimulated by nasal administration of chitosan; the immune response was sufficient to protect BALB/c mice from infection with the H7N9 virus, a highly relevant and pathogenic virus [360].

So far, most of the literature on nasal administration of chitosan-based formulations have focused on vaccine development, as summarized by Boroumand et al. [7].

## 7. General Considerations

### 7.1. Toxicity and Irritation

Generally, chitosan is considered nontoxic as well as biocompatible and biodegradable and already frequently utilized in delivery systems and scaffolds intended for topical administration [71]. Several products comprising chitosan are already approved by the U.S. Food and Drug Administration for certain applications [42], such as wound dressings and sponges with hemostatic properties and could be found on the market [361]. Among the various chitosan-based marketed products intended for the localized treatment, chitosan has been used in hemostatic bandages or gels and sprays for skin wounds in HemCon^®^ and ChitoClear^®^, respectively, and as gels and sprays for nasal mucosal membranes in, e.g., ChitoRhino. A more detailed overview of these chitosan-based products is presented by Matica et al. [19]. To our knowledge, there are no chitosan-based products intended for localized treatment of vaginal infections on the market. Nevertheless, chitosan exists in broad ranges of MWs, DDA and originate from various sources and should therefore be thoroughly assessed when utilized as a constituent [45]. Moreover, it is pivotal to ensure that no traces of impurities from these natural products are present in the end-product [361].

Many studies find no or negligible irritancy and adverse effects upon topical, localized application of chitosan in animal models after, e.g., skin [175,182,187], vaginal [322] or ocular administration [335,362]. Nevertheless, some studies have found certain negative effects of chitosan in the in vitro conditions. Wiegand and colleagues fond MW-dependent cytotoxicity of chitosan in keratinocytes. They reported highly reduced viability in cells treated with chitosan with MW of around 120 kDa after only two hours, while oligosaccharides caused reduced viability in higher concentrations after 24 h. The cell death seemed to be mediated by the activation of effector caspases three and seven [363]. Chatelet and colleagues found that chitosan, independent of DDA, inhibited the proliferation of fibroblasts, while chitosan of lower DDA had antiproliferative effects on keratinocytes. However, no cytotoxic effects were observed towards any of these cell lines [364]. These studies highlight the importance of meticulous assessments of the potentially adverse effects of chitosan. There are many important considerations upon preparing chitosan as delivery systems or scaffolds in localized therapy; however, most studies seem to deem chitosan relatively safe.

### 7.2. Limitations

Chitosan is an attractive and versatile polymer in biomedical applications. The extent of its applicability reflects in the extensive literature on chitosan-based delivery systems in pharmaceutical science. Nevertheless, compared to the multitude of research performed, limited chitosan products are commercially available; moreover, several challenges remain to be addressed regarding the clinical success of chitosan [5,75,361]. The naturally derived polymer is generally obtained by deacetylation of chitin from crustacean shells, and the animal origin entails bacterial and protein contamination [361]. The deacetylation of chitin will partly remove the contamination; however, not adequately regarding biomedical application [75]. Moreover, the presence of heavy metals, such as lead, and mercury limits the purity of chitosan [361]. The production and especially the purification of chitosan entail a considerable cost that might restrain the development of chitosan products. However, the lower cost and more basic purification methods for fungal chitosan are gaining increased interest [20]. Moreover, fungal chitosan is not considered animal-derived and avoids shellfish allergenic proteins, although, holds fungal-specific antigens. The attractive diversity of chitosan also introduces challenges; for instance, the varying DDA and lack of defined MW complicate the passing of regulatory rules [20]. Additionally, the comparison of performed research is intricate due to variations that can affect chitosan properties and activity, such as the antimicrobial activity of chitosan [75,365].

The formulation of chitosan-based delivery systems can be limited due to the poor solubility of chitosan [5]. The solubility of chitosan is dependent on the DDA, MW, and pH, and chitosan is only soluble under acidic conditions [17]. This limits the possibilities for application, but it can be advantageous for some routes, such as the vaginal route that holds a suitable acidic environment [8]. The low pH involves more positively charged chitosan that intensifies its antimicrobial activity; however, these pH conditions might harm cells or tissue [75].

Challenges restricting the applicability of chitosan, such as issues of purity, solubility, pH, stability and mechanical properties, need to be addressed. Moreover, the environmental impact associated with conventional chitin extraction and processing demands attention to assure the sustainability of large-scale chitosan production from an ecological point of view.

## 8. Outlook

Chitosan offers numerous advantages as both a pharmaceutical excipient and a material with intrinsic antimicrobial potential. We hope that a unique emphasis on its broader potential as an active building block for various superior formulations destined for localized antimicrobial therapy will highlight its importance in the current AMR era. Due to its superiority in bioadhesion and biocompatibility, its use in localized antimicrobial therapy should be further exploited. It can be tailored to formulate an optimal delivery system or a device addressing the specific challenges of the administration site such as the skin and wound infections, a mucosal site such as the vagina, ocular site, nasal, etc. Its potential in dental formulation needs to be further expanded. Moreover, its ability to act in synergy, enhancing the antimicrobial potential of active ingredients, requires more in-depth studies and mechanistic insights. However, it is important to address the challenges of its sources. The marine sources need to be utilized in an eco-responsible manner, assuring sustainability. A more green chemistry approach must be used in the modification of chitosan’s properties. In summary, the extended role of chitosan must be seen as a dynamic one. The enlisted evidence needs to be further exploited, the somewhat surprising findings need to be confirmed, and novel roles discovered. In an era of AMR, the urgency of carrying out these projects should be fairly apparent.

## Figures and Tables

**Table 1:** Chitosan-based delivery systems and scaffolds in biofilm eradication for localized antimicrobial therapy. The MW and DDA denoted for each study.

Delivery System/Scaffold	System	Microorganisms	Findings	Ref.
	MW			
	DDA			
Particles and carriers	CNPs: Chitosan 107 kDa DDA: 75–85%	*S. aureus* MRSA MRSE	In vitro antibiofilm activity: The CNPs were able to inhibit biofilm formation in all strains, however, only by around 30% for MRSE, while *S. aureus* and MRSA had inhibitions of more than 90% and approximately 75%, respectively.	[151]
	CNPs: Chitosan 1.6 kDa DDA: 92% Rhamnolipid	*S. aureus* *S. epidermidis* *Klebsiella oxytoca*	In vitro antibiofilm activity: CNPs with the surface-active compound rhamnolipid demonstrated superior antibiofilm activity against *S. aureus* and *S. epidermidis*; however, the antibiofilm action in the Gram-negative bacteria *K. oxytoca* was absent.	[152]
Excipient and coatings	Chitosan-coated NPs or CNPs incorporated into microneedles: Chitosan 50–190 kDa DDA: n.a. Doxycycline	*S. aureus* *P. aeruginosa*	In vitro antibiofilm activity: The bacterial eradication of doxycycline-loaded NPs with chitosan was superior to the free drug in all strains. More than 99% of the bacterial biofilm was eradicated at 4× MIC. Ex vivo porcine antibiofilm activity: the chitosan-coated NPs incorporated in microneedles eradicated upwards of 97% of the bacteria in all strains.	[153]
	Lipid-polymer hybrid nanovesicles: Chitosan ≈250 kDa DDA: 75–85% Vancomycin	MRSA	In vitro antibiofilm activity: The lipid-polymer hybrid nanovesicles with vancomycin demonstrated superior eradication of MRSA biofilm.	[154]
	Microneedles: Chitosan MW: n.a. DDA: n.a. Zinc	*E. coli* *S. aureus*	In vitro antibiofilm activity: The zinc-loaded microneedles displayed superior antibiofilm activity in a concentration-dependent manner. In the highest zinc concentrations, almost all bacteria were killed. The unloaded microneedles killed more bacteria than the control but were less effective than the zinc-loaded microneedles.	[155,156]
	Hydrogel: Chitosan LMW DDA: n.a. Methylene blue	*Propionibacterium acnes*	In vitro antibiofilm activity: The chitosan/poloxamer hydrogel displayed moderate but significant antibiofilm activity; however, no additional antibiofilm effects from methylene blue were observed.	[157]
	Composite matrix: Chitosan 50–190 kDa DDA: ≥85% Silver NPs	*C. albicans*	In vitro antibiofilm activity: Compared to the control, the chitosan matrix with silver NPs and silver NPs alone reduced the number of viable cells in both *C. albicans* strains.	[158]
	Film: Chitosan 120 kDa DDA: n.a. Ciprofloxacin	*S. aureus* *P. aeruginosa*	In vitro antibiofilm activity: The ciprofloxacin-loaded films comprising chitosan and bacterial cellulose eradicated bacteria within 1 h of treatment. Inhibition towards both strains; however, stronger in *P. aeruginosa*	[159]
Polymer-based gels	Hydrogel: Chitosan 25–35 kDa DDA: >90% Toluidine blue O	*S. aureus* *P. aeruginosa*	In vitro antibiofilm activity: The hydrogels comprising chitosan and HPMC with toluidine blue O displayed good anti-biofilm activity in biofilms produced by *S. aureus* or *P. aeruginosa* with 1- to 3-log bacterial killing and proper biofilm penetration.	[160]
	Hydrogel: Chitosan 320 kDa DDA: n.a. Antimicrobial peptides: (ASP)-1 ASP-2	*P. aeruginosa* *A. baumannii* MRSA	In vitro antibiofilm activity: The peptide-loaded hydrogels had strong anti-biofilm activity in *P. aeruginosa* and MRSA, especially in *P. aeruginosa,* where the formulation was superior to a commercialized silver product. Ex vivo porcine antibiofilm activity: The peptide-loaded hydrogels surpassed the commercial product in all three strains and exhibited a strong eradication of the biofilms.	[161]
	Hydrogel: Chitosan LMW DDA: 95.6% ε-poly-L-lysine	MDR- *P. aeruginosa* MRSA *C. albicans*	Ex vivo antibiofilm activity: The ε-poly-l-lysine loaded hydrogels reduced the thickness of the polymicrobial biofilm and reduced the bacterial load of *P. aeruginosa*; however, the bacterial burden of the other organisms was not reduced.	[162]
	Hydrogel: Chitosan 190–375 kDa DDA: n.a. Silver NPs	*P. aeruginosa* MRSA	Polymicrobial biofilm activity: The silver NP-loaded chitosan hydrogel significantly reduced the bacterial load of MRSA in all concentrations of the nanoparticles. The bacterial load of *P. aeruginosa* was also reduced; however, the reduction was lower than for MRSA, and higher NP concentrations were required.	[163]
	Hydrogel: Succinyl chitosan 200 kDa DDA: 87% Cellobiose dehydrogenase Cellulase from *Trichoderma longibrachiatum*	*S. aureus* *E. coli*	In vitro antibiofilm activity: The unloaded hydrogel demonstrated anti-biofilm activity against both *S. aureus* and *E. coli*. The enzyme-loaded hydrogel had approximately the same level of inhibition.	[164]
Scaffolds	Matrix: Chitosan 200 kDa or 350 kDa DDA: n.a. Papain	*S. aureus* *S. epidermidis*	In vitro antibiofilm activity: Slightly improved activity from MMW chitosan with papain compared to HMW chitosan.	[165]
	Membrane: Chitosan 311.5 kDa DDA: 71% Cis-2-decenoic acid Bupivacaine	MRSA	In vitro antibiofilm activity: Almost all membranes displayed significant antibiofilm effects both upon evaluating the growth on the dressings and in wells.	[166]

The n.a. refers to not applicable/not denoted; CNP: chitosan nanoparticles, DDA: degree of deacetylation, HMW: high molecular weight, LMW: low molecular weight, MDR: multi-drug-resistant MIC: minimum inhibitory concentration, MMW: medium molecular weight, HPMC: hydroxypropyl methylcellulose, MRSA: methicillin-resistant *S. aureus*, MRSE: Methicillin-resistant *S. epidermidis*, MW: molecular weight, NPs: nanoparticles.

**Table 2:** Chitosan-based delivery systems and scaffolds in in vivo wound healing and skin damage models intended for localized antimicrobial therapy.

Delivery System/Scaffold	System	Microorganisms	Findings	Ref.
	MW			
	DDA			
Particles and carriers	CNPs: Chitosan 50–190 KDa DDA: 75–85% Antimicrobial peptide LL-37	MRSA	MRSA-infected wound model in mice: No growth of MRSA was observed in the group treated with LL-37 loaded CNPs after 7 days. This antibacterial effect was superior to all other treatment conditions.	[64]
	CNPs: Chitosan 50–190 kDa DDA: n.a. Cefadroxil	*S. aureus*	*S. aureus*-infected wound model in rats: CNPs loaded with cefadroxil embedded in in situ poloxamer 407 hydrogel showed a significant reduction in the bacterial burden in the wounds and complete healing after 5 days.	[184]
	CNPs: Chitosan LMW DDA: 75–85% Vancomycin hydrochloride	MRSA	MRSA-infected wound model in mice: the rats treated with pH-responsive CNPs comprising gemini surfactants loaded with vancomycin displayed a significantly reduced bacterial burden compared with both drug-free CNPs and free vancomycin.	[185]
Excipient and coatings	Beads: Chitosan MW: n.a. DDA: 90% Zinc oxide NPs		Noninfected wound model in mice: The bacterial growth in the wound without induced infections treated with any of the chitosan/PVA/zinc beads was lower than the control. Almost no growth was observed in mice treated with chitosan, chitosan/PVA, or the loaded beads.	[176]
	Chitosan-functionalized graphene quantum dots: Chitosan oligosaccharide MW: n.a. DDA: n.a. Graphene quantum dots	*S. aureus*	*S. aureus*-infected wound model in rats: the rats treated with the chitosan-functionalized quantum dots composite together with illumination exhibited improved wound healing compared to all the other groups.	[186]
Polymer-based gels	Hydrogel: Chitosan LMW DDA: >85% Terbinafine hydrochloride	*C. albicans*	*C. albicans-*infected wound in rats: chitosan hydrogel loaded with vesicles comprising penetration enhancers produced a significant reduction of *C. albicans* in the wound bed.	[187]
	Hydrogel: Cyclodextrin-modified chitosan 15–22 kDa DDA: 75−80% Diclofenac Silver ions	*P. aeruginosa*	*P. aeruginosa*-infected wound in a murine model: β-cyclodextrin modified chitosan supramolecular hydrogel loaded with diclofenac and silver ions displayed improved wound healing and reduced the bacterial load in the wound bed.	[188]
	Hydrogel: Chitosan MW: n.a. DDA: ≥95% Silver nitrate Calcium sulfate dehydrate Zinc nitrate hexahydrate Copper nitrate trihydrate	*S. aureus*	*S. aureus*-infected wound model in mice: The chitosan/ion hydrogel in gauzes surpassed chitosan alone and the control group in wound healing. Additionally, the group treated with the chitosan/ion hydrogel in gauzes significantly reduced bacterial load in the wound bed compared with chitosan alone.	[189]
	Hydrogel: Chitosan 300–450 kDa DDA: 85–95% Silver sulfadiazine	*S. aureus*	*S. aureus*-infected burn and wound model in mice: The healing rate of the wounds treated with the silver sulfadiazine nanocrystal in the hydrogel was faster, and the overall healing was superior to a cream formulation in both the burn and wound model.	[190]
	Hydrogel: Chitosan MW: 100−150 kDa DDA: 85% Ciprofloxacin Fluconazole	*S. aureus* *E. coli* *C. albicans*	Polymicrobial wound model in rats: The ciprofloxacin and fluconazole-loaded fibrin NPs loaded in chitosan hydrogel bandage displayed a significant reduction in microbial load in the infected wound compared to the unloaded- fibrin NPs loaded in chitosan. However, there was still some microbial growth after 14 days.	[191]
	Hydrogel: Chitosan (maleic anhydride grafted chitosan) MW: n.a. DDA: n.a. Antimicrobial peptide Hydrogen peroxide	MRSA	MRSA biofilm-infected wound model in mice: The hydrogel loaded with antimicrobial peptide and hydrogen peroxide displayed a significant reduction in bacterial viability compared to all other treatments; however, not complete eradication. Chitosan alone reduced bacterial viability. Wound closure also improved in the groups treated with the coloaded hydrogel.	[192]
Scaffolds	Film: Chitosan 50–190 kDa DDA: 75–85% S-nitrosoglutathione	MRSA	MRSA biofilm-infected wound model in mice: Both loaded and unloaded chitosan films reduced the bacterial burden in the wound and improved the healing rate compared to the control group. However, the NO-releasing film displayed a significantly improved healing and dispersal of the biofilm.	[193]
	Film: Chitosan 200 kDa DDA: 85% Catechol	MRSA	MRSA-infected wound model in rats: The bacterial load in the group treated with the catechol-chitosan film at a reduced state was significantly reduced compared with the other groups. Additionally, the tissue in this group appeared normal.	[194]
	Film: Chlorinated chitosan MMW DDA: 75–85% Chloramine	MRSA	MRSA-infected wound model in mice: Chlorinated chitosan film produced with electrofabrication induced faster wound healing and reduced the wound’s bacterial burden, compared to the control and plain chitosan.	[195]
	Membrane: Chitosan MW: n.a. DDA: 87% Silver sulfadiazine	*P. aeruginosa,* *S. aureus*	*S. aureus* and *P. aeruginosa* infected-wound model in rats: The membranes significantly reduced the bacterial load in the wounds compared to the control group with a rapid initial eradication.	[196]
	Dressing: Chitosan 190–310 kDa DDA: 75–85% Silver NPs	*P. aeruginosa*	*P. aeruginosa*-infected wound model in mice: The mice treated with the polyelectrolyte complex had a reduced bacterial load in the tissue after 14 days of treatment and higher survival than mice treated with gauze.	[197]
	Nanofibers: Chitosan ≈250 kDa DDA: n.a. Indocyanine green	MRSA	MRSA-infected wound model in rats: The indocyanine green-loaded chitosan/PVA nanofibers and illumination demonstrated improved wound healing and reduced bacterial burden in the wound bed compared to all other treatment groups.	[198]
	Dressing loaded with microspheres: Chitosan MMW DDA: ≥85% Gentamycin sulfate	*S. aureus* *E. coli*	*S. aureus* and *E. coli*-infected wound model in rats: The group treated with gelatin microspheres loaded with gentamycin and platelet-rich plasma on chitosan dressing displayed reduced bacterial load and a faster wound healing rate than the group treated with gauze.	[199]
	Dressing: Chitosan MMW DDA: 97% Graphene oxide Copper NPs	*S. aureus*	*S. aureus*-infected wound model in mice: The group treated with the graphene oxide–copper composite in chitosan/hyaluronic acid hydrogel improved wound healing compared with all other groups.	[200]
	Sponge: Chitosan 10–30 kDa DDA: ≥95% Silver NPs	*S. aureus*	*S. aureus*-infected wound model in rabbits: the group treated with the silver NP-sponge healed faster than the control group, and although not statistically significant, faster than the marketed silver dressing.	[201]
	Sponge: Chitosan MW: 500 kDa DDA: 90% Quaternary ammonium CNPs	*S. aureus*	*S. aureus*-infected wound model in mice: the chitosan sponges loaded with quaternary ammonium CNPs exhibited superior antimicrobial activity compared to sponges without CNPs and untreated mice on days 7 and 10.	[202]

n.a.: not applicable/not denoted; CNP: chitosan nanoparticles, DDA: degree of deacetylation, LMW: low molecular weight, MMW: medium molecular weight, MRSA: methicillin-resistant S. aureus, MW: molecular weight, PVA: poly(vinyl alcohol).

**Table 3:** Antimicrobial activity of chitosan-formulations targeting vaginal infections.

Formulation/Role of Chitosan	Targeted Vaginal Infection	Active Ingredient	Main Finding(s)	Ref.
Particles and carriers	Candidiasis	Clotrimazole	Decreased antifungal activity in vitro but improved safety profile for CNP-associated clotrimazole	[285]
		Callophycin A	Synergetic and improved antifungal effect both in vitro and in vivo by Callophycin A in CNPs	[286]
		Thiosemicarbazide	CNP-associated thiosemicarbazide obtained a prominent reduction of fungal burden in vivo	[287]
		Miconazole	CNPs containing a seven-fold lower miconazole concentration than conventional miconazole cream obtained equal therapeutic effect	[288]
		Miconazole and farnesol	The combination of miconazole and farnesol in CNPs expressed a greater antifungal effect in vivo	[289]
		Argentinean medicinal plants	Chitosan microcapsules containing active substances exhibited strong antifungal capacity in vitro	[290]
	Bacterial vaginosis	Doxycycline	CNP-associated doxycycline expressed a significant reduction in *E. coli* viability in vitro	[291]
		Metronidazole	Superior in vitro inhibition of *B. fragilis* growth by chitosan-alginate microspheres containing metronidazole	[265]
	Aerobic vaginitis	Cefixime	Cefixime microspheres reduced *E. coli* viability in vitro	[292]
	Trichomoniasis	-	CNPs expressed concentrations and time-dependent antimicrobial activity against *T. vaginalis* in vitro	[293]
	Gonorrhoea	-	CNPs expressed antigonoccocal activity against all tested strains, including high-level resistant *N. gonorrhoeae*	[294]
Coating material and excipient	Candidiasis	Tioconazole and econazole	Chitosan-coated nanocapsules maintained the antifungal activity for both drugs in vitro	[295]
		Clotrimazole	Chitosan-coated PLGA NPs increased the antifungal activity of clotrimazole in vitro	[296]
		Metronidazole	Fungal inhibition was equal for the chitosan-based formulation containing metronidazole and the drug-free formulation	[27]
		Herbal medicine	All constituents of the microemulsion expressed antifungal activity in vitro, including chitosan	[297]
		Metronidazole	In vitro antifungal activity was increased in the presence of chitosan and independent of metronidazole	[298]
		Amphotericin B	The hydrogel containing amphotericin B-loaded chitosan microplatelets obtained a complete cure of infection in vivo	[299]
		Fluconazole	Chitosan-based vaginal inserts containing fluconazole showed improved antifungal activity both in vitro and in vivo compared to free drug	[300]
		Curcumin	Curcumin liquid crystal system containing chitosan increased the antifungal potency of curcumin in vitro	[301]
			Curcumin liquid crystal system containing chitosan significantly decreased fungal burden in vivo and efficiently reduced the growth of biofilm in vitro	[302]
		Fluconazole	Chitosan-based vaginal tablets increased the antifungal activity of fluconazole	[303]
		*Chelidonii herba* extract	Chitosan-based vaginal tablets detained the in vitro antimicrobial activity of the extract	[304]
		Ketoconazole	Ketoconazole-containing chitosan and gellan gum gel flakes in thermosensitive gel expressed antifungal effect in vivo	[305]
		Itraconazole	Thermosensitive gel with a chitosan gel-flake system significantly improved the antifungal effect of itraconazole in vivo	[306]
		Chlorhexidine	Chitosan-based vaginal inserts increased the antifungal activity of chlorhexidine in vitro	[307]
			Chitosan-based vaginal inserts increased the antifungal activity of chlorhexidine in vitro	[308]
	Bacterial vaginosis	-	Chitosan-coated liposomes expressed in vitro antibacterial effect against *S. epidermidis* and *S. aureus*	[8]
		Metronidazole	Vaginal tablets containing chitosan and metronidazole inhibited *B. fragilis* growth in vitro	[309]
	Aerobic vaginitis	Chlorhexidine	Chitosan-based vaginal inserts increased the antimicrobial activity against *E. coli* of chlorhexidine in vitro	[307]
			Chitosan-based vaginal inserts increased the antimicrobial activity against *E. coli* of chlorhexidine in vitro	[308]
		Metronidazole	Metronidazole vaginal tablets containing chitosan exhibited in vitro antimicrobial effect against *E. coli*	[310]
	Trichomoniasis	-	In vitro antimicrobial effect was related to the PIBCA NPs and dependent on chitosan coating of NPs	[271]
		Metronidazole	Increased in vitro antimicrobial activity of chitosan-coated NPs compared to noncoated	[270]
			Antimicrobial effect of metronidazole was maintained when in chitosan delivery system	[311]
		-	The hydrogel containing chitosan proved to reduce *T. vaginalis* motility in biological fluids	[312]
Vaginal gel	Candidiasis	-	Chitosan hydrogel was confirmed to have intrinsic antifungal properties in vitro	[313]
		*Thymbra capitata* essential oil	Chitosan hydrogel with essential oil showed increased in vitro antifungal activity and the ability to disrupt biofilm in a dose-dependent manner	[314]
		*Mitracarpus frigidus* extract	Chitosan hydrogel with the extract obtained antifungal effect in vivo comparable to the marketed product	[315]
		Miconazole or econazole	Superior in vitro antifungal activity by LMW chitosan hydrogel containing miconazole	[316]
		*Pelargonium graveolens* essential oil	Chitosan hydrogel-thickened nanoemulsion containing essential oil expressed antifungal activity in vitro	[317]
		-	Chitosan hydrogel significantly reduced biofilm formations both in vitro and in an in vivo model	[318]
		Iminoboronate	Fungicidal activity in biomimetic conditions and inhibition of biofilm formation in vitro was obtained	[319]
	Bacterial vaginosis	-	Low concentration chitosan hydrogel efficiently eradicated *Pseudomonas aeruginosa* biofilms in vitro	[24]
			Superior in vitro activity against *S. aureus* and *S. epidermidis* by chitosan formulated as hydrogel	[8]
		Metronidazole	3D printed vaginal ring containing chitosan and metronidazole obtained a bactericidal effect against *E. coli* and confirmed synergistic antibacterial effect by chitosan and metronidazole	[320]
	Trichomoniasis	Auranofin	The chitosan-based hydrogel containing auranofin NPs managed to completely inhibit parasite growth in vitro in a dose-dependent manner	[321]
	Chlamydia	Resveratrol	Superior antichlamydial activity in vitro was by resveratrol liposomes-in-hydrogel in the lower concentrations	[276]
Vaginal film	Candidiasis	Fluconazole	The chitosan-based vaginal film obtained in vitro fungal growth inhibition comparable to the marketed product	[322]
		Tioconazole	Drug-free chitosan vaginal film expressed in vitro fungicidal activity; however, superior activity when loaded with tioconazole	[323]
		Econazole	Chitosan-based matrices containing econazole microparticles expressed antifungal activity in vitro	[324]
	Bacterial vaginosis	Metronidazole	Chitosan-based membrane did not restrain the effect of metronidazole against *S. aureus* and *G. vaginalis* in vitro	[325]
	Aerobic vaginitis	Ciprofloxacin	Vaginal films enhanced the activity of ciprofloxacin against *E. coli* and *S. aureus* in vitro	[326]

CNPs: chitosan nanoparticles, NPs: nanoparticles, PIBCA: poly(isobutylcyanoacrylate), LMW: low molecular weight, MPs: microparticles.

## Data Availability

Data sharing not applicable.
